# Inoculation Improves Microbial Manganese Removal during the Start-Up of Rapid Sand Filters

**DOI:** 10.1021/acsestwater.5c00050

**Published:** 2025-04-15

**Authors:** Signe Haukelidsaeter, Alje S. Boersma, Thilo Behrends, Wytze K. Lenstra, Niels A. G. M. van Helmond, Lina Piso, Frank Schoonenberg, Paul W. J. J. van der Wielen, Maartje A. H. J. van Kessel, Sebastian Lücker, Caroline P. Slomp

**Affiliations:** † Department of Earth Sciences, Faculty of Geosciences, Utrecht University, P.O. Box 80021, 3508 TA Utrecht, The Netherlands; ‡ Department of Microbiology, Radboud Institute of Biological and Environmental Science, Faculty of Science, Radboud University, P.O. Box 9010, 6500 GL Nijmegen, The Netherlands; § Vitens N.V., P.O. Box 1205, 8001 BE Zwolle, The Netherlands; ∥ KWR Water Research Institute, P.O. Box 1072, 3430 BB Nieuwegein, The Netherlands

**Keywords:** drinking water production, rapid sand filtration, filter replacement, microbial manganese oxidation, nitrification

## Abstract

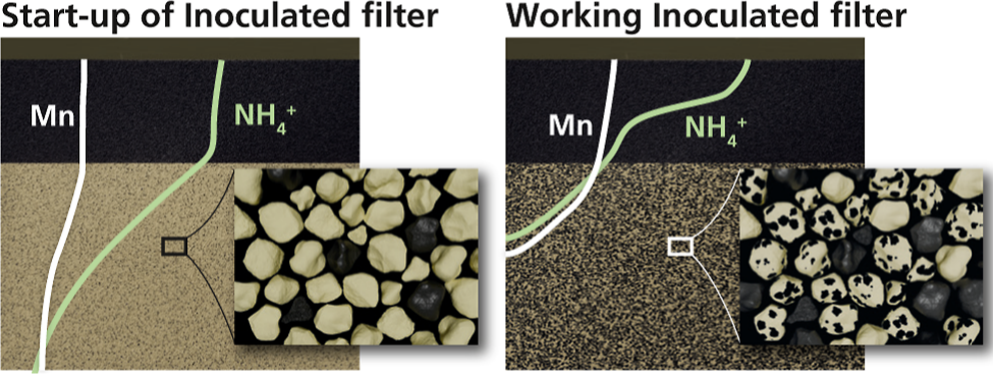

Long start-up times to achieve manganese removal in rapid sand filters can pose challenges for drinking water companies. This study assessed the start-up dynamics of manganese removal in two full-scale dual-media rapid sand filters treating groundwater containing iron, ammonium, and manganese. After inoculation with 20% biologically active coated sand, ammonium and manganese removal efficiencies of ∼60–70% and ∼30–50% were achieved, respectively. Complete removal of ammonium occurred after ∼8 weeks, but ∼17 and ∼25 weeks were required for manganese removal in the two filters. Full manganese removal, accompanied by manganese oxide formation on new grains, was achieved when ∼50% of the ammonium was removed within the anthracite layer. X-ray spectroscopy of manganese oxides in the mineral coatings indicated a dominance of biologically produced manganese oxide with a structure similar to that of δ-MnO_2_, suggesting continuous microbial manganese oxidation in inoculated rapid sand filters. Concomitant changes in 16S rRNA gene profiles combined with qPCR and solute profiles suggest a key role for *Nitrospira* in both nitrification and manganese oxidation. We show that inoculation with biologically active filter medium enhances the efficiency of ammonium and manganese removal during filter start-up, offering a promising improvement strategy for rapid sand filters.

## Abstract 2

Inoculation of rapid sand filters improves ammonium and manganese removal, potentially due to increased *Nitrospira* abundance.

## 1 Introduction

Globally, around 50% of drinking water is produced from groundwater, which often contains dissolved manganese(II), henceforth referred to as manganese.^1^ Manganese must be removed to prevent drinking water discoloration, metallic taste, health issues, and the potential of clogging in distribution systems caused by the oxidation and precipitation of solid manganese oxides.^2,3^ Aeration followed by rapid sand filtration is one of the oldest and safest methods for manganese removal from groundwater.^4^ However, every time a new rapid sand filter is started, incomplete manganese removal occurs, and establishing full removal can take months to years, posing challenges for drinking water companies.^3,5^

The initial phase of manganese removal in rapid sand filters is predominantly driven by microbial activity^6^ and can be impacted by the presence of iron and ammonium.^7,8^ A phylogenetically diverse group of heterotrophic microorganisms, for example, *Leptothrix*, *Hyphomicrobium*, and *Pseudomonas*, is suggested to be involved in manganese oxidation.^4,9,10^ While *Leptothrix* spp. might also play a role in iron oxidation, *Gallionella* are the dominant chemolithoautotrophic iron-oxidizing bacteria.^11^ Iron often occurs in higher concentrations in groundwater than manganese,^12^ and because of the rapid chemical and biological oxidation with oxygen, iron is removed faster than manganese in the top part of the filter.^2,7^ If iron is removed homogeneously in the water, the resulting iron flocs can inhibit biological manganese oxidation,^13^ which thus often occurs deeper in the filter.^14^

The presence of ammonium may also have an effect on the removal of manganese, but the reason for this is less well understood. Ammonium can be removed either in a two-step process by ammonia-oxidizing and nitrite-oxidizing bacteria or by comammox *Nitrospira*, which perform both steps.^15^ In rapid sand filters, *Nitrospira*, comprising comammox and canonical nitrite-oxidizing *Nitrospira*, are often the dominant nitrifiers^9^ and are found together with ammonia-oxidizing *Nitrosomonas* species.^16,17^ Effluent data from newly started rapid sand filters show that complete ammonium removal takes weeks to establish, and complete manganese removal is often achieved months to years later.^18−20^ Vertical solute profiles show that nitrification either precedes or occurs concurrently with manganese removal.^14,19^ Both direct involvement of nitrifiers in manganese oxidation^20^ and indirect effects of nitrification on manganese oxidation (O_2_ removal, pH decline, nitrite accumulation^22^) have been suggested. Still, the exact mechanisms that may control this interaction remain unclear.

Manganese(IV)-phylomanganates, the product of microbial manganese oxidation, will slowly form a filter medium coating.^19,21^ This mineral coating can initiate abiotic, heterogeneous manganese oxidation, causing precipitation of new manganese oxides.^6,23^ Moreover, the coating will enhance the attachment of microorganisms, which, in turn, will increase the removal of both ammonium and manganese in the filter.^19,24^ Adding biologically active, coated filter medium can improve manganese removal during start-up, as demonstrated in column studies treating manganese-containing but iron and ammonium-free groundwater.^25,26^ However, the impact of inoculating a full-scale sand filter treating groundwater containing iron, manganese, and ammonium with biologically active manganese-coated sand during the start-up phase has not yet been assessed.

In this study, we inoculated the sand layer of two newly started dual-media rapid sand filters with 20% manganese-coated sand at a drinking water treatment plant (DWTP) in The Netherlands. This DWTP processes anoxic groundwater rich in iron, manganese, and ammonium. The goal of this study was to evaluate the filter performance after inoculation with manganese-oxide-coated sand and improve our understanding of microbial ammonium and manganese removal in the start-up phase of rapid sand filters.

## 2 Materials and Methods

### 2.1 Drinking Water Treatment Plant

At the Sint Jansklooster DWTP in The Netherlands (52°40′41.2″N, 6°00′47.8″E), groundwater is extracted from 18 wells across two main source areas. The source water has a pH of 6.9–7.0 and a temperature of 10–11 °C, with iron, manganese, ammonium, and methane concentrations varying based on the combination of wells in use (Supporting Information). The treatment process consists of 7 steps, starting with plate aeration and oxygen gas dosing, followed by cascading aeration of the water to the primary rapid sand filter. Subsequent steps include intensive tower aeration, pellet softening, pH correction by CO_2_ dosing, secondary rapid sand filtration, and ion exchange to remove natural organic matter (Figure S1).

This study focuses on the primary rapid sand filtration step using gravitational filters, which mainly removes iron, manganese, ammonium, and residual methane not removed by plate aeration. The DWTP operates 12 parallel dual-media primary rapid sand filters, each with a surface area of 25 m^2^, containing 0.9–1.0 m of anthracite (clean bed: 1.4–2.5 mm diameter, ∼50% porosity) and 1.6 m of sand (clean bed: 0.8–1.2 mm diameter, ∼42% porosity). The supernatant height ranges from 10 to 25 cm, and the filtration flow varies from 45 to 65 m^3^/h, depending on production demand.

The aeration steps prior to sand filters are essential to ensuring sufficient oxygen (O_2_) to support ammonium removal. Plate aerators undergo monthly cleaning to prevent iron buildup, thus O_2_ concentrations can vary in the supernatant. Additionally, pure oxygen dosing was implemented in 2021 to ensure O_2_ concentrations remain above 400 μM (>14 mg/L) in the supernatant. This adjustment was necessary due to the high ammonium concentrations in some extraction wells, which could exceed 220 μM (>4 mg/L).

Filters are backwashed every 3–4 days. The process uses ∼200 m^3^ of prefiltrate water and follows this sequence: a 3 min water wash at 1500 m^3^/h, a 2 min gradual water wash reducing from 1500 to 0 m^3^/h, a 5 min air scour at 60 N m^3^/m^2^/h, a 4 min water wash at 1500 m^3^/h, and a final 1 min 40 s gradual wash reducing from 1500 to 0 m^3^/h.^27^

The filter medium of the rapid sand filters at this location is replaced every 8–10 years because of failing manganese removal.^19^ Historical data show that with conventional replacement without inoculation, most iron is removed immediately after filter replacement (Figure S2). Complete ammonium removal can take 6–8 weeks to establish, whereas complete manganese removal occurs after 12–21 weeks (Figure S2). Excess iron, manganese, and ammonium remaining in the primary filter effluent are removed by a secondary filter.

In this study, we present data in both text and figures in μM to directly visualize the relationship between the solutes involved in the chemical reactions in the filter. We refer to the supplement for the corresponding values in mg/L.

### 2.2 Filter Replacement

Two primary filters, Filter 1 (VF11) and Filter 4 (VF14), were replaced and put in operation on the 28th of February and 26th of April 2023, respectively. The coated sand used as the inoculant was removed from the bottom (approximately the lowest 30 cm) of the replaced filters, corresponding to around 7.5 m^3^ coated sand. Given that the sand layer is fully mixed during backwash,^27^ the sand layer is expected to be homogeneous throughout with respect to its chemical and microbial composition. The grain size of the inoculum was marginally coarser (by about 5 to 24 μm) compared to new fresh sand, but the overall shape remains quite similar between the inoculant and the new sand material (Supporting Information). The removed material was stored for 3 days in covered containers under wet conditions to avoid contamination and drying of the filter medium (Figure S3A).^26^

The filters were filled with 130 cm of new sand (32.5 m^3^) and backwashed (Figure S3B). Subsequently, a 30 cm layer of the stored coated sand was added as an inoculum (∼7.5 m^3^) per filter. The sand was pumped onto the filters directly from the containers and was then distributed evenly across the filter surface by backwashing (Figure S3C, D). Subsequently, 90 cm of fresh anthracite was added to Filter 1 (Figure S3E), and 70 cm of anthracite was added to Filter 4. The filters were backwashed again to remove dust and small particles, which resulted in the complete mixing of the new and coated sand. The sand used as inoculum had a distinct black color compared to the new material, which consisted of white/glassy grains without manganese coating (Figure S3F).

### 2.3 Sample Collection and Analysis

Water and filter medium samples were collected weekly during the first 4 weeks of operation, biweekly for the next three months, and monthly for the subsequent 2 months.

Groundwater samples were collected from a tap before aeration. To assess iron, manganese, ammonium, and methane removal over the filter, samples were collected from 12 taps along the side of Filter 1 and 4 taps along Filter 4, allowing vertical profiles over the filter depth. Effluent samples were taken from the outlet pipe draining the filter. Because of the higher depth resolution of Filter 1 compared to Filter 4, the results from Filter 4 are presented in the Supporting Information.

Oxygen (O_2_) concentrations, pH, conductivity, and temperature were measured directly from the taps using an HQ40D Portable Multimeter (Hach), with a tube from each tap directing water into a continuously overflowing 500 mL polypropylene bottle.

Filtered (0.45 μm nylon syringe filter, Genetec) water samples were collected in 15 mL polypropylene centrifuge tubes, acidified with ultrapure nitric acid (HNO3; 10 μL per 1 mL sample), and stored at 4 °C until analysis for iron and manganese. The iron and manganese concentrations were assumed to represent dissolved iron and manganese(II). Additionally, filtered water samples were collected for ammonium, nitrite, and nitrate determination and stored at −20 °C until analysis. For methane, 100 mL glass bottles were filled from the bottom up and allowed to overflow; bottles were closed with butyl rubber stoppers and crimped with aluminum caps. The samples were directly poisoned with saturated zinc chloride and stored upside down in the dark at room temperature.

Dissolved iron (limit of detection, LOD = 0.4 μM) and manganese (LOD = 0.018 μM) were analyzed by using a PerkinElmer Avio 500 inductively coupled plasma optical emission spectrophotometer. The concentrations of ammonium (LOD = 0.3 μM), nitrite (LOD = 0.02 μM), and nitrate (LOD = 0.09 μM) in the water samples were determined spectrophotometrically with a Gallery Discrete Analyzer. Ammonium, nitrite, and nitrate were measured according to ISO7150-1:1984. To determine methane, 10 mL of nitrogen gas was added as headspace to the samples while simultaneously removing the same amount of water. Methane was analyzed using a Thermo Finnigan TraceTM gas chromatograph with a flame ionization detector (LOD 0.02 μM).

Removal percentages across filters were calculated by comparing the concentrations of iron, manganese, and ammonium in the supernatant layer above the filter to the concentrations in the effluent below the filters from each sampling time. The effluent is representative of the removal of the entire filter bed. Percentage removal for all samplings is presented in the Supporting Information

Filter medium samples were collected to a depth of 2.4 m with a stainless-steel peat sampler. In Filter 1, four separate depth samples were taken from the anthracite layer (0–90 cm) and six from the sand layer at 20 cm intervals (100–240 cm). In Filter 4, three samples came from the anthracite layer (0–70 cm) and seven from the sand layer (80–240 cm) at 20 cm intervals. About 40 to 50 mL filter medium per sample was collected in 50 mL centrifuge tubes and were stored at −20 °C.

### 2.4 Geochemical Analysis of Filter Medium

For high-resolution imaging and elemental mapping, sand (depth of 150–175 cm) from day 1, week 6, week 19, and week 30 for Filter 1 and day 1 and week 22 for Filter 4 was analyzed using a Zeiss Evo 15 scanning electron microscope with energy-dispersive X-ray spectroscopy (SEM-EDS; Method S1). Resin-embedded samples from the same depth, collected on day 1 and week 30 for Filter 1 were examined using μ-XRF and μ-X-ray absorption spectroscopy at the ID21 beamline at the European Synchrotron Radiation Facility in Grenoble, France, in January 2024 (Method S2).^28^ The analysis focused on extended X-ray absorption fine structure (EXAFS) and X-ray adsorption near edge structure (XANES).

### 2.5 Microbial Community Analysis

DNA was extracted from the filter medium for 16S rRNA gene amplicon sequencing (Method S3) and bacterial 16S rRNA gene qPCR analysis (Method S4).

## 3 Results and Discussion

### 3.1 Start-Up Time of Inoculated Full-Scale Rapid Sand Filters

**Figure 1:**
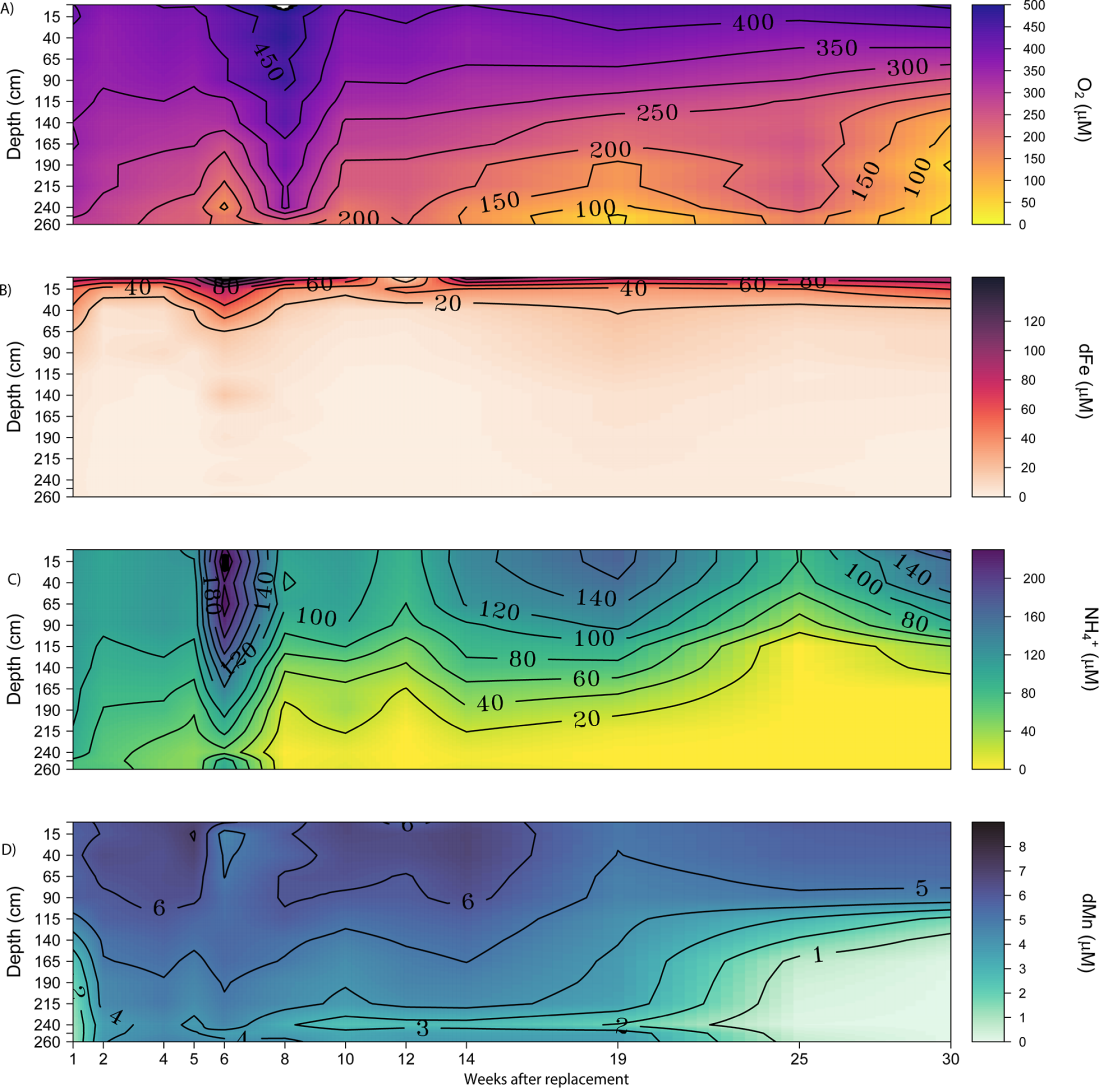
Dynamics of key solutes in Filter 1 following inoculation with manganese-coated sand (week 0) until complete filter function (week 30). (A) Oxygen (O_2_), (B) dissolved iron (dFe), (C) ammonium (NH_4_^+^), and (D) dissolved manganese (dMn) across the depth of the filter. The samples were collected 1 day and 1, 2, 4, 5, 6, 8, 10, 12, 14, 19, 25, and 30 weeks after filter replacement. Samples were collected between March and September 2023.

**Figure 2:**
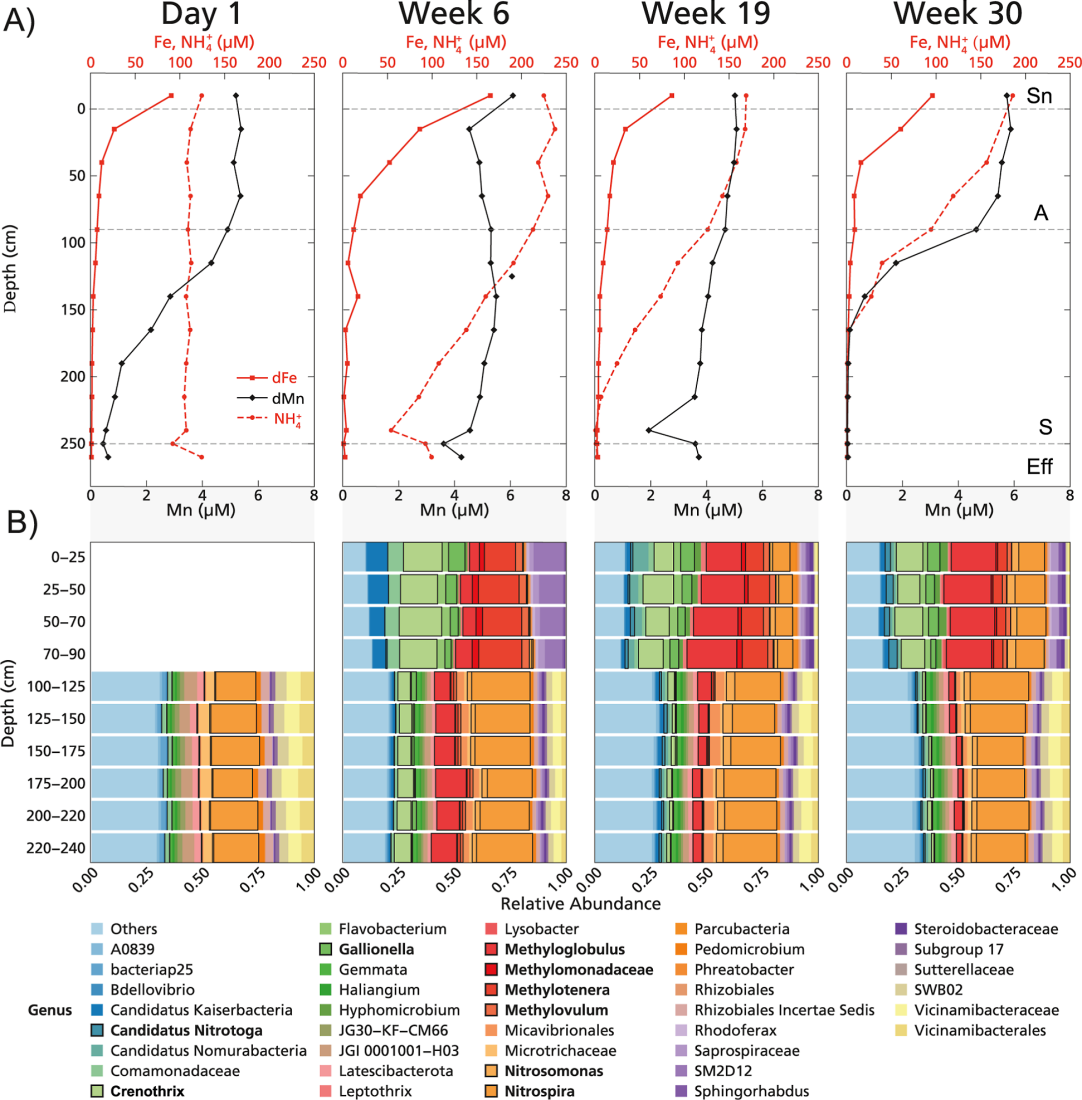
Removal profiles for solutes with depth combined with relative microbial community abundance on 1 day and 6, 19, and 30 weeks after filter replacement; (A) removal of iron, manganese, and ammonium with depth in Filter 1 in the weeks following filter replacement. Profiles for dissolved iron (dFe, red squares), dissolved manganese (dMn, black diamonds), and ammonium (red dotted line). Sn, supernatant; A, anthracite; S, sand; and Eff, effluent. Data for the other time points and Filter 4 are included in Figure S5 and the Supporting Information. (B) Microbial community composition based on 16S rRNA gene amplicon sequencing for Filter 1. Samples from 1 day and 6, 19, and 30 weeks after filter replacement were analyzed. Highlighted are key players in methane, iron, and ammonium removal (*Candidatus* Nitrotoga, *Crenothrix*, *Gallionella*, *Methyloglobulus*, *Methylomonadaceae*, *Methylotenera*, *Methylovulum*, *Nitrosomonas*, and *Nitrospira*).

Dissolved oxygen was always present at concentrations >30 μM throughout Filter 1 (Figure 1A). Iron was present in the supernatant at relatively high concentrations (∼100–170 μM), yet over 95% of dissolved iron was removed in the anthracite layer from day 1 onward (Figures 1B, 2A, and S4). *Gallionella*, known for their role in biological iron oxidation,^11^ were observed in the anthracite from week 6 onward at a relative abundance of 3 to 7% (Figure 2B) but were less abundant in the sand (relative abundance of 0.3% on average). About 3–13 μM of the dissolved iron leaked into the sand (Supporting Information), and structures resembling biologically formed iron oxides were observed in the sand layer (Figure S6).

During the weeks following filter replacement, most oxygen removal occurred in the sand layer, coinciding with the filter depths where nitrification mainly occurred (Figure 1A,C). Ammonium concentrations in the supernatant water ranged from 78 to 237 μM. In the first 4 weeks, 68–74% of the ammonium was removed in the inoculated sand layer through conversion to nitrate (Figures 1 and S7). In week 6, the groundwater well combination resulted in particularly high ammonium concentrations (>230 μM). At this time point, nitrification commenced in the anthracite layer of the filter, although most ammonium was removed from the sand (Figures 1C and S7). The relative abundance of *Nitrospira* and *Nitrosomonas* in the anthracite in week 6 (0.3–0.6% and 0.1–1.0%, respectively) was low; however, bacterial numbers were about 200 times higher in the anthracite compared to the sand (Figure S8). In absolute numbers, *Nitrospira* and *Nitrosomonas* were therefore more abundant in the anthracite compared to the sand, which is in accordance with the observed ammonium removal in the anthracite. Ammonium removal could be inhibited by the presence of iron flocs,^29^ which may explain the incomplete ammonium removal in the anthracite layer.

Eight weeks after inoculation, ammonium removal reached >90% efficiency, with nitrification occurring over the entire filter depth across both the anthracite and sand layer. However, most ammonium removal occurred in the sand layer until week 25(Figures 1 and S7). Between weeks 19 and 30, the relative abundance of *Nitrospira*, *Nitrosomonas*, and Candidatus *Nitrotoga* almost doubled in the anthracite layer, and the absolute bacterial quantity increased by a factor of 5 (Figures 2B and S9). Interestingly, considering the *Nitrosomonas* abundances determined for weeks 6, 19, and 30, the apparent ammonium conversion rate per cell decreases with time (Table S1). Furthermore, the relative abundance of *Nitrospira* in the sand layer (21–26%) remained high after week 30, even though more ammonium (50%) was removed in the anthracite layer (Figure 2).

Filters 1 and 4 showed a comparable pattern in microbial community development and solute removal (Figures 2, S4, S5, and S12). After 1 day, 88% of the incoming manganese was removed, but this decreased to ∼30% in the following weeks for Filter 1 and to ∼50% for Filter 4. The initially high manganese removal observed in both filters, followed by a sharp decline in removal efficiency in the following week, suggests an initial abiotic removal process that was not sustainable over time. This decline may indicate a transition to biologically mediated removal, which becomes inhibited by other processes in the filter. There is minor removal and release of manganese at the bottom of the filter (240 cm depth) between weeks 6 and 19 (Figures 1 and S4), but this has no effect on its overall functioning. Complete manganese removal was again observed after weeks 25 and 17 for Filters 1 and 4, respectively, and only occurred when ∼50% of the ammonium was removed in the overlaying anthracite layer (Figures 1D, 2, and S5). Directly after inoculation, the relative abundances of *Hyphomicrobium* and *Pedomicrobium*, both of which have been associated with manganese removal in rapid sand filters,^30,31^ were 1.3–1.6% and 2.0–2.6% in the sand, respectively (Figure 2B). The change to complete manganese removal did not coincide with an increased relative or absolute abundance of *Hyphomicrobium* and *Pedomicrobium* (Figure S10), suggesting that other microorganisms are responsible for manganese removal.

The residual methane (1–3 μM) present in the water after plate aeration was oxidized in the filter (Figure S11). Despite the relatively low concentrations of methane, a high relative abundance of *Crenothrix*, *Methyloglobulus*, *Methylomonadaceae*, *Methylotenera*, and *Methylovulum*, which are known to be involved in methane and methanol oxidation, was observed (Figure 2B). In the first 10 weeks, methane was removed in the anthracite and partly in the sand (Figure S11). However, after 10 weeks, all methane was removed in the anthracite layer, and consequently, the relative abundance of the methane-oxidizing community in the sand decreased (Figure 2B). After week 12, no methane entered the filter.

Overall, the microbial communities in Filters 1 and 4 exhibited clear changes over time, which coincided with changes in the removal of the various solutes. The microbial communities of both filters also became increasingly similar (Figure S13).

### 3.2 Manganese Oxide Characteristics Support Microbial Manganese Oxidation

**Figure 3:**
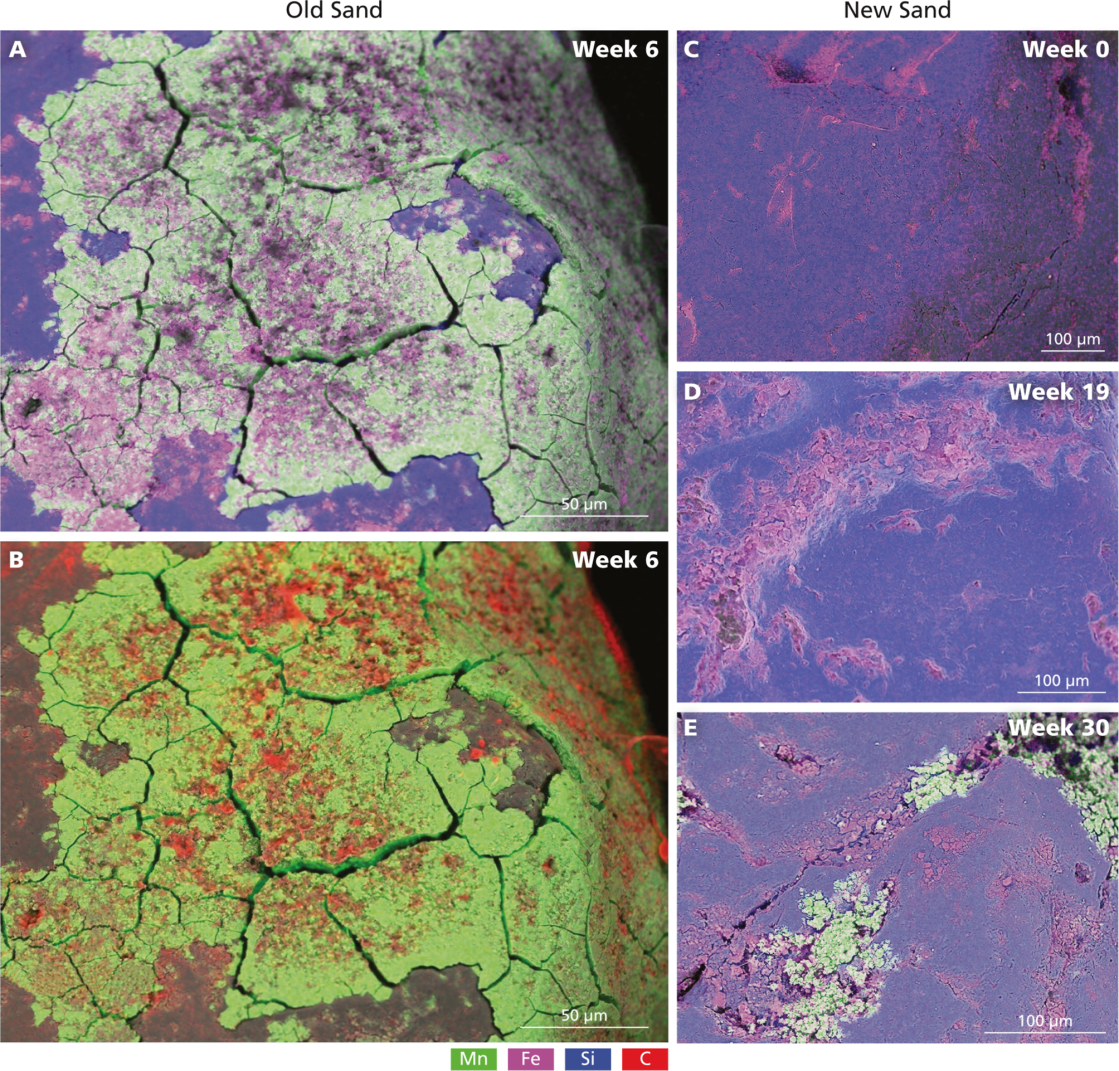
SEM-EDS images of filter medium collected at 125 to 150 cm depth from Filter 1. (A,B) Coated sand collected 6 weeks after filter replacement but originating from the inoculum. (C–E) New filter medium collected (C) on day 1, (D) at 19 weeks, and (E) at 30 weeks after filter replacement. Manganese (Mn) is shown in green, iron (Fe) in pink, silicon (Si) in blue, and carbon (C) in red. Images for Filter 4 are shown in Figure S15.

The manganese-coated sand that was used as an inoculum had a 5–15 μm thick coating of primarily manganese and iron oxides (Figure 3A). The coated sand also contained carbon, likely in the form of biofilm and adsorbed organic carbon, which was mainly associated with iron oxides (Figures 3, S6, and S14). This supports the importance of mineral coatings as attachment sites for microorganisms and organic carbon in sand filters.^19,24^

After just 1 day of operation, iron accumulation was already observed on the new (uncoated) sand grains (Figures 3 and S15). New manganese coatings were observed on new sand particles only after 30 weeks for Filter 1 (Figure 3) and after 22 weeks for Filter 4 (Figure S15). This suggests that the bacterial species involved in manganese oxidation needed time to establish on the new grains. This process was not accelerated by the addition of the coated filter medium as inoculant, when compared to the start-up times of uninoculated filters at this DWTP (Figure S2). The fresh manganese oxides primarily formed in the irregularities of the filter material on top of the already accumulated iron, which likely provided a rougher surface where coating and biofilm could develop (Figures S16 and S17). This formation began as small, isolated deposits that eventually grew to a more extensive manganese oxide coating (Figure 3).

**Figure 4:**
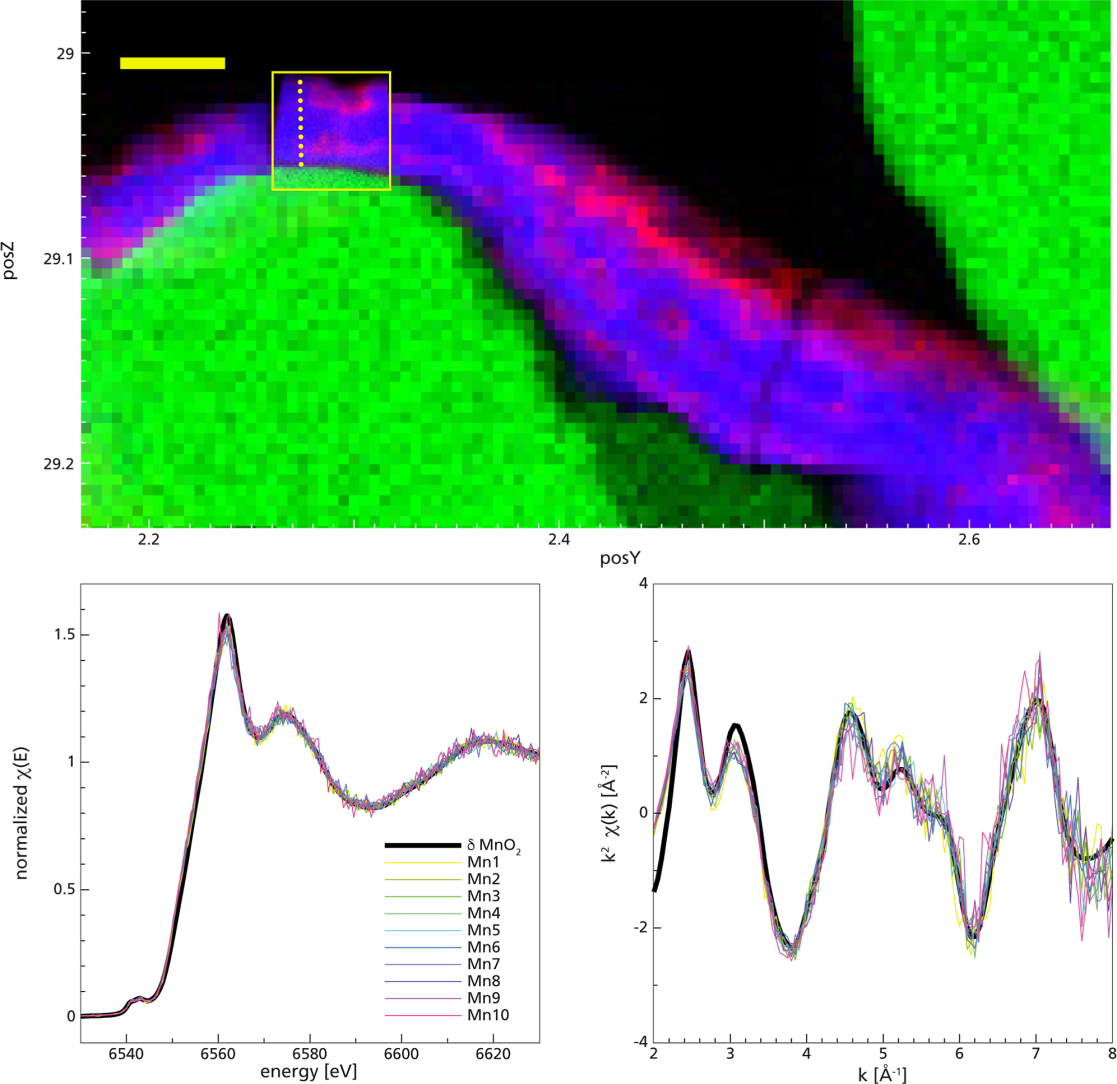
μ-XRF map of a resin-embedded and polished section of a coated sand grain retrieved from Filter 1 at 30 weeks after filter replacement; (A) distribution of manganese (blue), iron (red), and silicon (green). The scale bar represents a length of 50 μm. The large map was collected with a step size of 5 μm, while the map inside the yellow rectangle was mapped with a resolution of 0.5 μm. The yellow spots indicate positions at which XANES spectra were collected at the manganese K-edge. (B) Self-absorption manganese K-edge XANES (left) and (C) EXAFS (spectra) across the coating of a sand grain, compared to the spectrum of d-MnO_2_ reported by Webb et al. (2005)^33^ shifted by 0.4 eV. The corresponding positions of the spots are indicated in (A) with manganese 1 at the surface of the coating and manganese 10 at the interface between the coating and sand grain.

The self-absorption-corrected, normalized manganese XANES and EXAFS spectra collected along the cross sections of the coatings of sand collected after 30 weeks indicate a mineralogically homogeneous coating (Figure 4). The spectra closely resemble those of manganese precipitates formed by *Pseudomonas putida*, with a structure identified as δ-MnO_2_.^32^ The shape of the pre-edge and the position of the absorption maximum in the spectra from the coating are identical with those of δ-MnO_2_. The average manganese oxidation state is 3.84 when shifted by −0.4 eV, which is in the range of the energy resolution.^33^ Furthermore, no difference could be recognized between spectra collected along the cross sections of coatings present on the sand added as an inoculum and those after 30 weeks of operation (Figures S18 and S19). The spectroscopic characterization indicates that the manganese coatings consist of phylomanganates formed through microbial oxidation. The coatings mainly contain manganese(IV) but also some manganese with a lower oxidation state. The missing positive charge is likely balanced by Ca^2+^ based on the close correlation between calcium and manganese in the coating (Figure S20).

There are no indications that freshly formed manganese precipitates differ from the initial coatings, regarding their structure or oxidation state. The latter is unexpected as the substantial (88%) removal of manganese during the first day following filter replacement points toward adsorption of manganese(II), possibly followed by heterogeneous oxidation leading to manganese(III)-containing solids. However, the amount of manganese retained during the first 24 h of operation (∼4.7 × 10^–7^ g manganese g sand^–1^) reflects such a minor fraction of the manganese in the coating, that it is difficult to detect in XANES spectra. Alternatively, the adsorbed and potentially abiotically oxidized manganese transformed to manganese(IV), as manganese(III) is metastable and can react further to manganese(IV) phylomanganates under the release of manganese(II).^34^ In any case, these results show that microorganisms play an important role during the start-up of manganese removal in rapid sand filters and likely continue to play a key role in manganese removal in “mature” filters.

### 3.3 Possible Involvement of *Nitrospira* in Manganese Removal

Nitrification and manganese removal in rapid sand filters appear interdependent, particularly during the start-up phase.^5^ We found increased manganese removal in the sand layer only after 50% of the ammonium had been removed in the overlying anthracite layer (Figures 1 and 2A). Reduced oxygen concentrations and nitrite accumulation caused by nitrification can diminish manganese oxidation.^20,22^ This is not the case here since oxygen concentrations were lowest when ammonium and manganese removal was most efficient (Figure 1). Nitrite accumulation also did not affect manganese removal in the filters we studied (Figure S21). As noted above, the increase in removal efficiency did not align with an increased abundance of key manganese-oxidizing bacteria (Figures 2 and S10), suggesting that other microorganisms might be responsible for manganese oxidation.

A potential explanation for the observed interconnectivity of ammonium and manganese removal would be the involvement of nitrifiers in manganese oxidation. *Nitrospira* are the main ammonia oxidizers in these sand filters, and manganese oxidation by distinct members within the *Nitrospirota* has been demonstrated.^35^ Given that the metabolic yield of complete ammonia oxidation is higher than that of manganese oxidation (−318 vs −68 kJ mol^–1^, respectively^15,35^), comammox *Nitrospira* will preferentially oxidize ammonium. Based on the 16S rRNA gene sequencing results (Figure 2B), however, we cannot determine the proportion of comammox versus canonical *Nitrospira*. A recent metagenomic analysis of the filter material used for filter inoculation here revealed a roughly equal distribution between canonical and comammox *Nitrospira*, and in similar, noninoculated filters of similar age, canonical *Nitrospira* constituted most of the *Nitrospira* community.^36^ Since the energy yield from oxidizing nitrite to nitrate (−74 kJ mol^–1^^15^) is comparable to the yield of manganese oxidation, the latter represents a plausible alternative metabolism which could sustain the observed *Nitrospira*.

Moreover, if the high abundances of *Nitrospira* present in the sand layer (21–26%) were solely involved in nitrite oxidation, a similarly high number of ammonia oxidizers would be expected as canonical *Nitrospira* rely on ammonia oxidizers for nitrite. However, besides the presumably low number of comammox *Nitrospira*, *Nitrosomonas* was the only known ammonia oxidizer in the filters, and their relative abundance was only 2–3% during this period. Strikingly, the *Nitrospira* MAGs identified in the filter inoculum encoded the manganese oxidase MnxG and the outer membrane c-type cytochrome Cyc2,^36^ which is hypothesized to be involved in initial manganese oxidation in the *Nitrospirota* member *Candidatus* Manganitrophus noduliformans.^35^ Additionally, one *Nitrospira* MAG also encoded moxA, another protein involved in manganese oxidation.^36^ This supports a possible role of *Nitrospira* in manganese oxidation in these filters, which should be further tested, preferably by isolating *Nitrospira* strains from rapid sand filters and testing their potential for manganese oxidation.

### 3.4 Implications for Drinking Water Treatment and Future Research

Studying the start-up of full-scale rapid sand filters is important to test and validate results from lab- or pilot-scale column experiments under operating conditions. Column studies using inoculated filter material reported complete manganese removal from day one onward.^25,26^ However, this was not achieved in the full-scale filters investigated here. This may be explained by differences in the groundwater composition. The groundwater in our study contained iron, manganese, and ammonium, whereas the groundwater in those pilot studies contained manganese only.^25,26^ Still, despite the apparent inhibitory effects of ammonium on manganese oxidation, we find that the addition of biologically active coated sand results in increased manganese and ammonium removal when compared to filters that are not inoculated.^5,18,19^

At the DWTP studied here, the large water production volumes (∼60 m^3^/h per filter) and the manganese concentration of 5–6 μM (0.27–0.34 mg/L) can lead to a high amount of manganese passing through the filter in the weeks following start-up. For instance, with an estimated start-up time of 25 weeks for Filter 1, a total breakthrough of up to ∼100 kg of manganese can be expected. The inoculation with biologically active manganese-coated sand improved this removal efficiency by ∼30% (corresponding to ∼30 kg less Mn breakthrough) and by 50% in Filter 4 in 17 weeks (corresponding to ∼34 kg less Mn breakthrough), thereby reducing the required manganese removal capacity of the subsequent treatment steps.

Although the inoculation procedure was similar for Filters 1 and 4, the better performance of Filter 4 compared to Filter 1 can have multiple reasons at an operating plant with numerous variables that change with time and between filters. These include slightly different volumes of coated sand, differences in raw water quality, and filtration rates. Overall, both filters exhibited similar trends, with initial partial manganese removal and complete removal occurring only upon increased nitrification rates in the anthracite layer (Figures S4 and S5).

Potential negative effects of using inoculation on the filter’s longevity need to be assessed in future studies. However, based on the results from this study, it is likely that using a slightly larger volume of coated sand (25–30%) than the 20% we used here will improve the manganese removal and nitrification efficiency in the first weeks. Inoculating coated and biologically active anthracite in a filter treating high concentrations of iron is not recommended, however, due to the fragility of the anthracite and its coatings compared to the sand, which could compromise the longevity of the filter. Since new iron and manganese oxides are primarily formed within sand grain irregularities (Figures 3, S11, and S12), filter material with rougher surfaces could provide better attachment sites for metal oxide precipitation and microorganisms, promoting biofilm development and potentially contributing to a shorter start-up period.

Adopting less intense backwashing and slower water filtration rates during the filter start-up phase can provide better conditions for microbial attachment, coating formation, and shorter start-up of rapid sand filters.^37^ Applying an inoculation strategy with coated, biologically active sand in secondary polishing filters is promising^38^ as iron is already removed, reducing the need for regular backwashing. However, such an approach might result in higher biomass release to the drinking water as these secondary filters are often the last treatment step before the water is distributed as drinking water to the consumers. Since the filters investigated here also remove iron at high concentrations (<100 μM), regular backwash is needed, highlighting the complexity of balancing the removal of iron, manganese, and ammonium within one filter.

## 4 Conclusions

This study demonstrates that inoculation of rapid sand filters with biologically active coated filter sand can enhance both manganese and ammonium removal during filter start-up, thereby reducing the load of these compounds in subsequent treatment stages. However, the start-up time to ensure complete removal was not reduced as a result of inoculation. We show that manganese removal in these filters is primarily microbial and interacts with other biogeochemical processes, particularly nitrification. Manganese is only removed after ammonium is removed more in the filter. The high abundance of *Nitrospira* at depths where manganese is microbiologically removed, along with the reported presence of manganese oxidase genes in their genomes, suggests a potential role for *Nitrospira* in manganese oxidation in these rapid sand filters.

The findings emphasize the importance of microbial processes for both manganese and ammonium removal in rapid sand filters for treating anoxic groundwater. Future research should investigate the role of *Nitrospira* in manganese oxidation in rapid sand filters and assess the effects of inoculation with coated sand on filter longevity. Additionally, investigating filter media with rougher surfaces and higher concentrations of inoculant may offer improvements for microbial attachment and manganese oxidation rates, potentially further enhancing the manganese removal efficiency of rapid sand filters during start-up.

## Supporting Information Available

Supplementary methods, supplementary figures, and calculated rates of ammonium removal per nitrifier cell in the filter (PDF)
Chemical data collected from Filter 1 and Filter 4 at different time points, 16SrRNA, qPCR, and grain size analysis (XLSX)
