# Supplementary material for: Inoculation Improves Microbial Manganese Removal during the Start-Up of Rapid Sand Filters
Source: ACS ES T Water. 2025 Apr 15;5(5):2479–89. doi: 10.1021/acsestwater.5c00050 (PMC12070406; doi:10.1021/acsestwater.5c00050)
Supplement: Supplementary file 1 — ew5c00050_si_001.pdf [file ew5c00050_si_001.pdf]

Inoculation improves microbial manganese removal during start-up of rapid sand filters

Signe Haukelidsaeter[1*], Alje S. Boersma[2*], Thilo Behrends[1], Wytze K. Lenstra[2], Niels A.G.M van Helmond[1], Lina Piso[2], Frank Schoonenberg[3], Paul W.J.J. van der Wielen[2,4,5], Maartje A.H.J. van Kessel[2], Sebastian Lücker[2†], Caroline P. Slomp[1,2†]

Supplementary Information:

This file contains:

- Supplementary Methods S1-S4
- Supplementary Figures S1-S19
- Supplementary Table S1
- Supplementary Data (provided as a separate Excel file)

Supplementary methods:

Method S1: For geochemical analyses of the filter medium, sand samples collected at 125-150 cm filter depth from Filter 1 on day 1, and weeks 6, 19, and 30 after filter replacement were chosen. Samples were air-dried, platinum-coated, and analyzed using a Zeiss Evo 15 Scanning electron microscopy with energy dispersive X-ray spectroscopy (SEM-EDS) to obtain high-resolution images with elemental mapping.

Method S2: Resin-embedded samples from 150-175 cm filter depth, from day 1 and week 30 from Filter 1 and Day 1 and Week 22 for Filter 4 were further investigated using μ-XRF and μ-X-ray absorption spectroscopy at beamline ID21 (Salomé et al., 2013) at the European Synchrotron Radiation Facility (ESRF) in Grenoble, France, in January 2024. The beam was focused using KB mirrors to the size of about 1000 × 560 nm^2 (horizontal × vertical). XRF maps were collected at 7200 eV with a step size (vertically and horizontally) of either 5 or 1 mm. At selected spots, X-ray absorption spectra at the Mn K-edge were collected in fluorescence mode in the energy range 6.50-6.90 keV. XRF maps were deconvoluted and corrected for the intensity of the incoming beam (I0) using the software PYMCA (Solé et al., 2007). X-ray absorption spectra were processed using the software Athena (Ravel & Newville, 2005). Many X-ray spectra collected on spots in the coating showed features indicative of self-absorption. The amplitude of the pre-edge in normalized spectra was higher compared to reference spectra collected in transmission mode and the white line intensity was considerably lower. Therefore, self-absorption correction was applied using the XANES(FLUO) self-absorption correction module in Athena. For this, the generic composition was assumed to be MnO_2 × n H_2O, and n was adjusted to obtain similar amplitudes for the pre-edge and the white line as the reference spectrum of the JMn-1 Mn nodule (Terashima et al., 1995).

Method S3: DNA was extracted from 0.5 g (wet weight) of filter material using the Dneasy PowerSoil DNA Isolation Kit (QIAGEN, Hilden, Germany), with minor modifications to the manufacturer's protocol. Cell lysis was achieved by bead beating at 50 Hz for 1 minute using a TissueLyser LT (QIAGEN, Hilden, Germany). In cases where DNA yield was insufficient, up to three lysis replicates, each using 0.5 g of sample material, were pooled onto a single GeneJet Spin column. DNA was eluted in 50 μL of DEPC-treated water following a 1-minute incubation of the silica matrix in the DEPC water at room temperature before the final centrifugation. For samples used for qPCR, DNA was always isolated separately using 0.5 g

of filter medium (wet weight). The dry-to-wet-weight ratio was later determined by incubating 1 g of filter medium in an oven at 60 ºC for 24 h and measuring the weight loss.

16S rRNA gene sequencing was conducted by Macrogen Inc. (Seoul, South Korea) using the Illumina MiSeq platform. Bacterial 16S rRNA genes were amplified using primers 341F (5′-CCTACGGGNGGCWGCAG-3′;Herlemann et al., 2011) and 806R (5′-GGACTACHVGGGTWTCTAAT-3′; Caporaso et al., 2012)Paired-end libraries were prepared with the Herculase II Fusion DNA Polymerase and Nextera XT Index Kit V2 (Illumina, San Diego, USA) following the 16S Metagenomic Sequencing Library Preparation protocol (Part # 15044223 Rev. B). Between 81,000 and 142,000 paired-end reads were obtained per sample. Data processing was performed in R (v3.5.1; R Core Team, 2019) using the DADA2 pipeline (v1.8;Callahan et al., 2016). Taxonomic classification of 16S rRNA gene sequences was based on the SILVA database (release 138.1;Quast et al., 2013). Relative abundances, as calculated by DADA2, were analyzed using the Phyloseq package (v1.30.0;McMurdie & Holmes, 2013) in R.

Method S4: Bacterial 16S rRNA gene copy numbers were quantified using qPCR. Amplification of the 16S rRNA gene was performed with primers 331F (5′-TCCTACGGGAGGCAGCAGT-3′; Nadkarni et al., 2002) and 518R (5′-ATTACCGCGGCTGCTGG-3′; Muyzer et al., 1993). gBlocks gene fragments (Integrated DNA Technologies, Leuven, Belgium) of the target genes were used as standards at concentrations ranging from 6.26×10^8 to 6.26×10^3 copies/µL. PCR reactions were prepared in a total volume of 10 µL, consisting of 5 µL Quanta mix (Bio-Rad Laboratories B.V., Veenendaal, The Netherlands), 3 µL DNase-free water, 0.5 µL of 10 µM forward and reverse primer in DNase-free water, and 1 µL of DNA template. Reactions were run on a C1000 Touch thermocycler equipped with a CFX96 Touch real-time detection system (Bio-Rad Laboratories B.V., Veenendaal, The Netherlands). The PCR program consisted of an initial denaturation at 95 °C for 5 minutes, followed by 40 cycles of denaturation at 95 °C for 30 seconds, annealing at 60 °C for 30 seconds, and elongation at 72 °C for 15 seconds. Subsequently, a melting curve from 60 °C to 95 °C with 0.5 °C increments every 5 s was recorded. First, copy numbers were calculated per gram dry weight filter medium. Then, this was converted into copy numbers per cubic meter using the bulk densities of the used anthracite (680 kg m^{-3}) and sand (1500 kg m^{-3}).

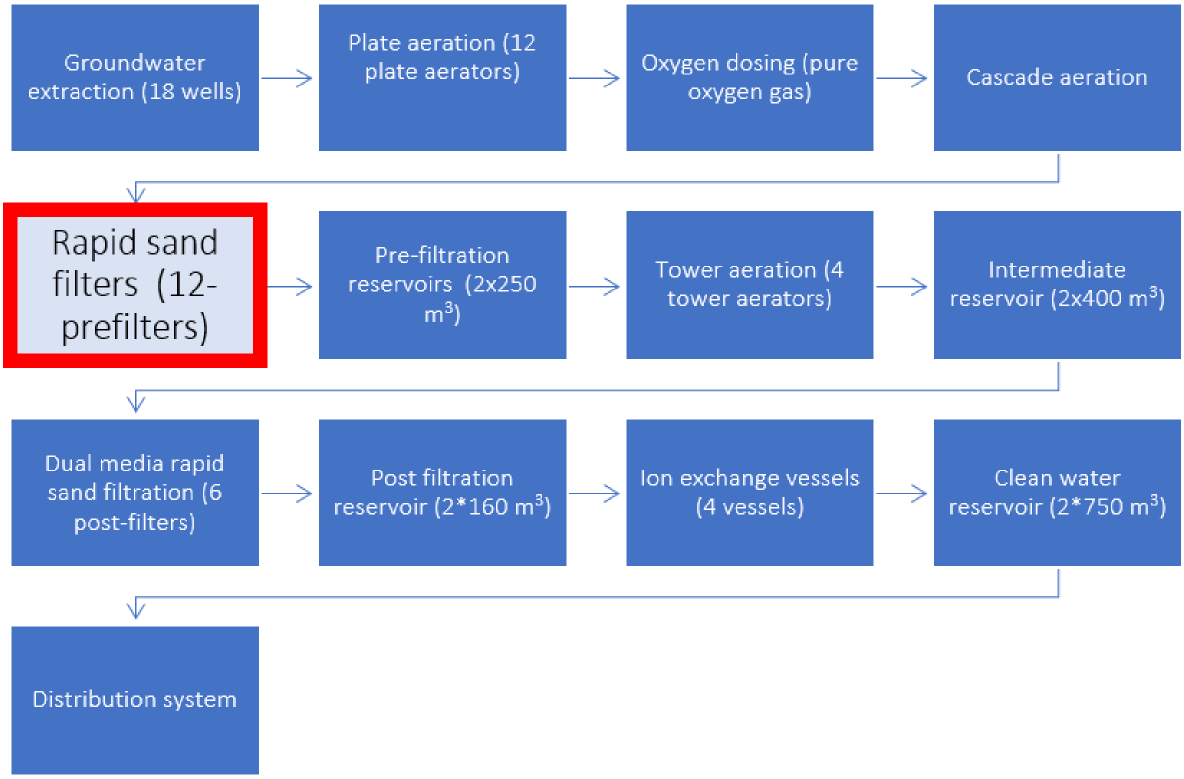

Figure S1. Treatment scheme at the Sint Jansklooster DWTP. The dual-media rapid sand filtration step (highlighted) is the treatment step analyzed in this study.

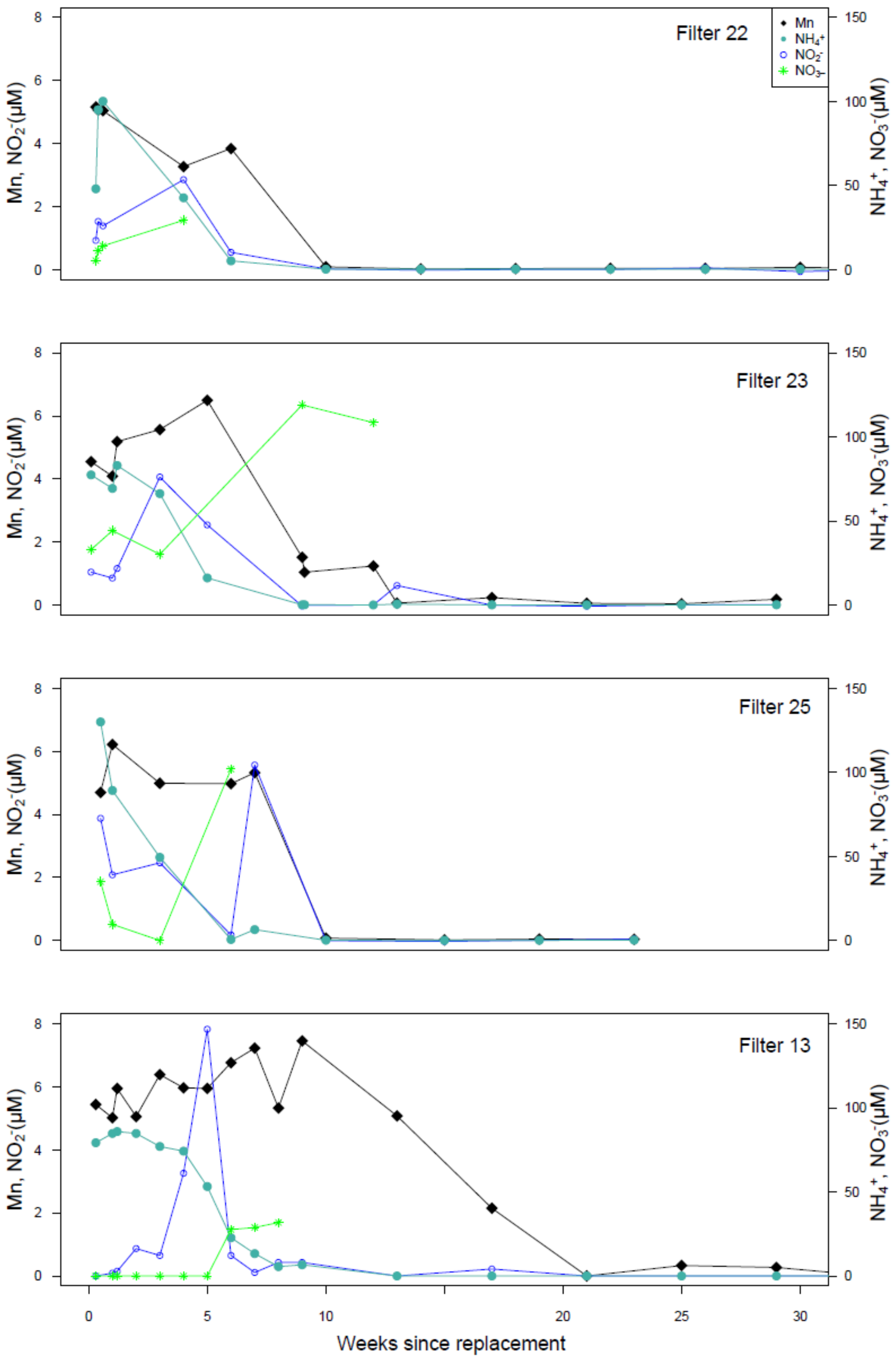

Figure S2. Historical effluent data from conventional filter replacement using only groundwater as inoculant. Concentrations of manganese (black diamonds) and ammonium (green dots), nitrite (blue open circles), and nitrate (light green stars) after replacement of Filter 22 (26.11.2021), Filter 23 (20.05.2022), Filter 25 (05.07.2022), and Filter 13 (01.03.2021).

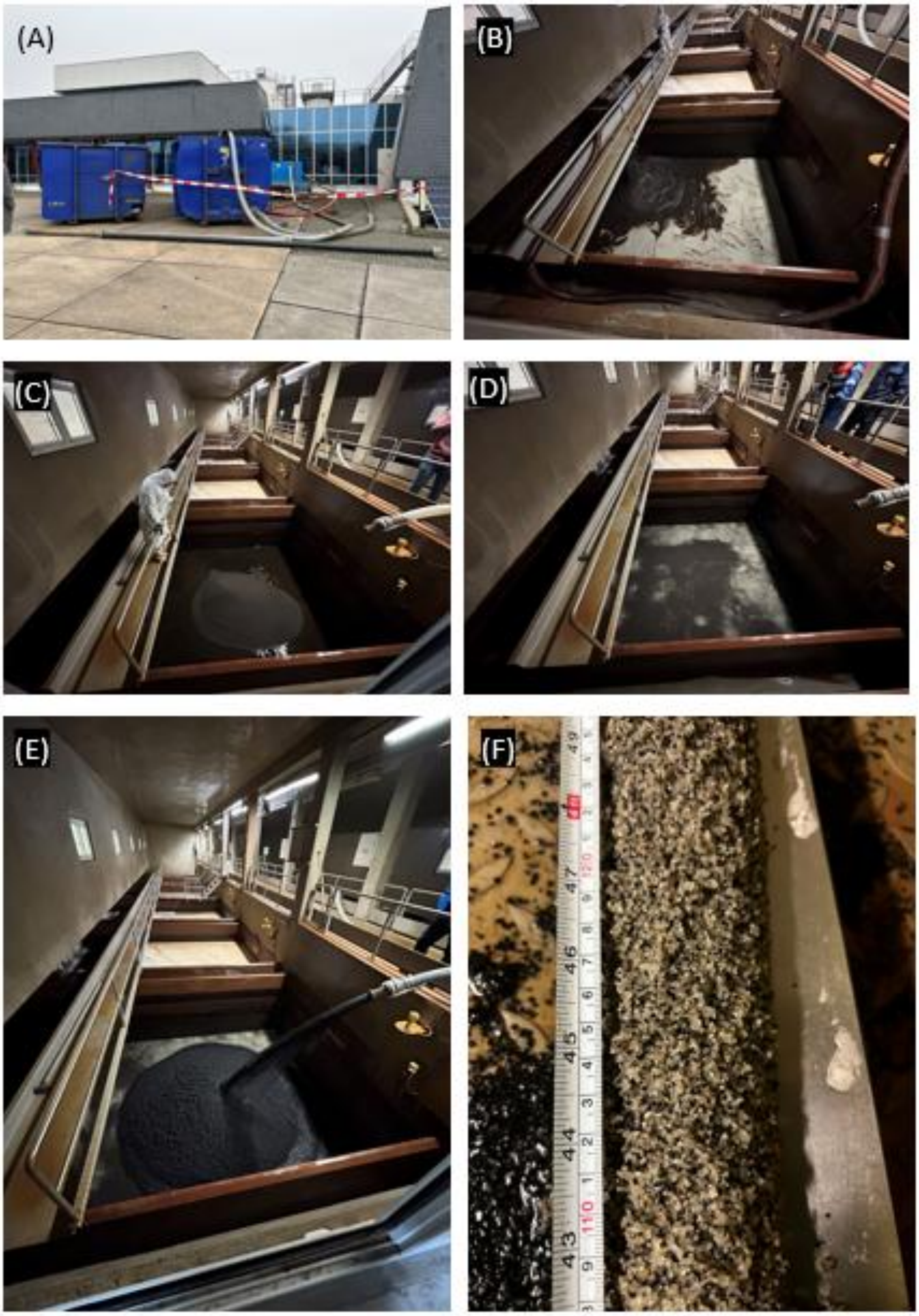

Figure S3. Photographs of the replacement and inoculation of Filter 1 in March 2023. A) Storage container for manganese oxide-coated sand. B) Addition of manganese oxide-coated sand on top of the new sand. C) Pile of manganese oxide-coated sand in the middle of the filter. D) Sand after backwashing with air to flatten the manganese oxide-coated layer. D) Addition of anthracite. F) Mixture of new and coated sand after a second backwash.

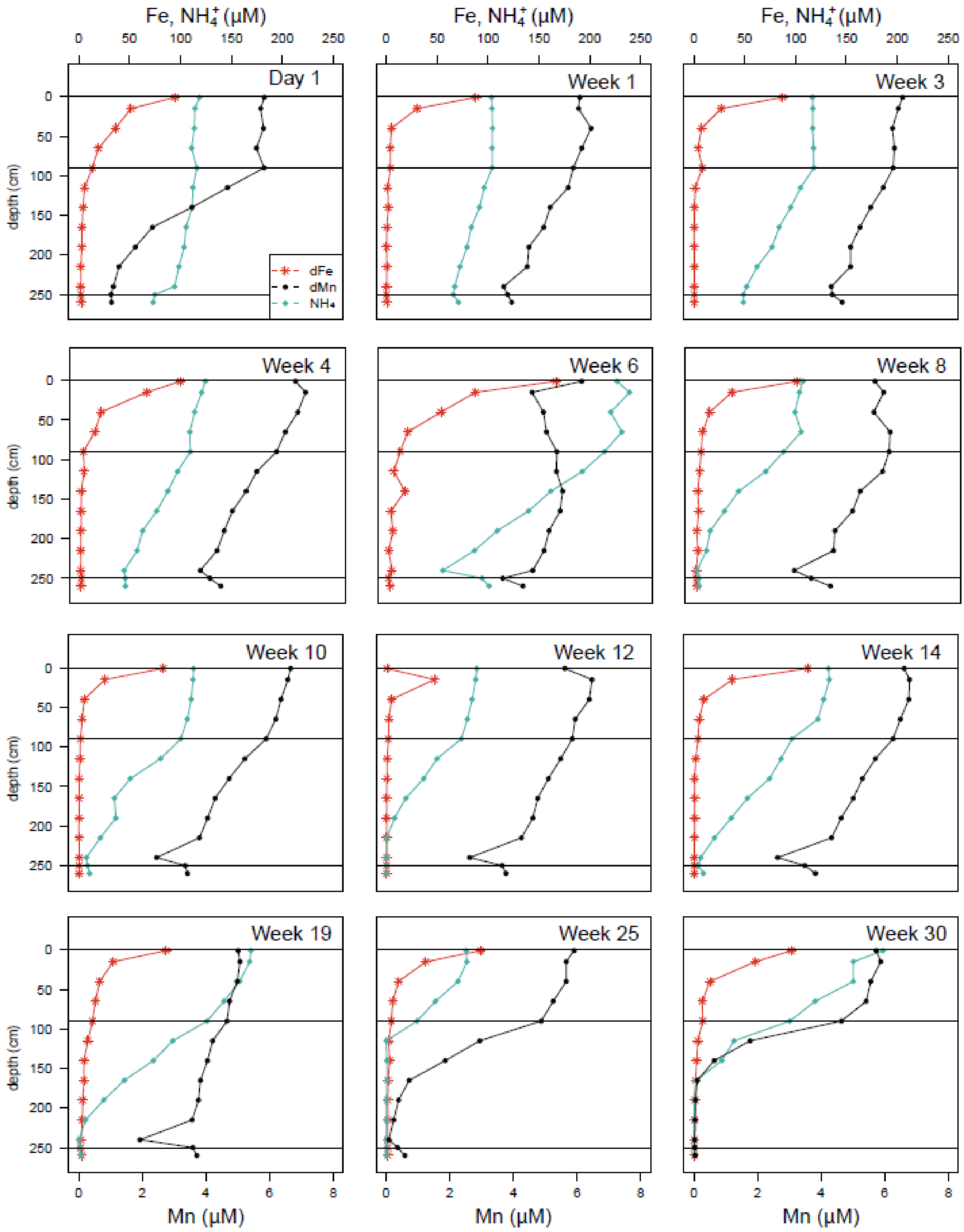

Figure S4. Temporal dynamics of dissolved iron (dFe, red stars), dissolved manganese (dMn, black diamonds), and ammonium (NH_4^+, light green diamonds) at 12 time points after inoculation of Filter 1.

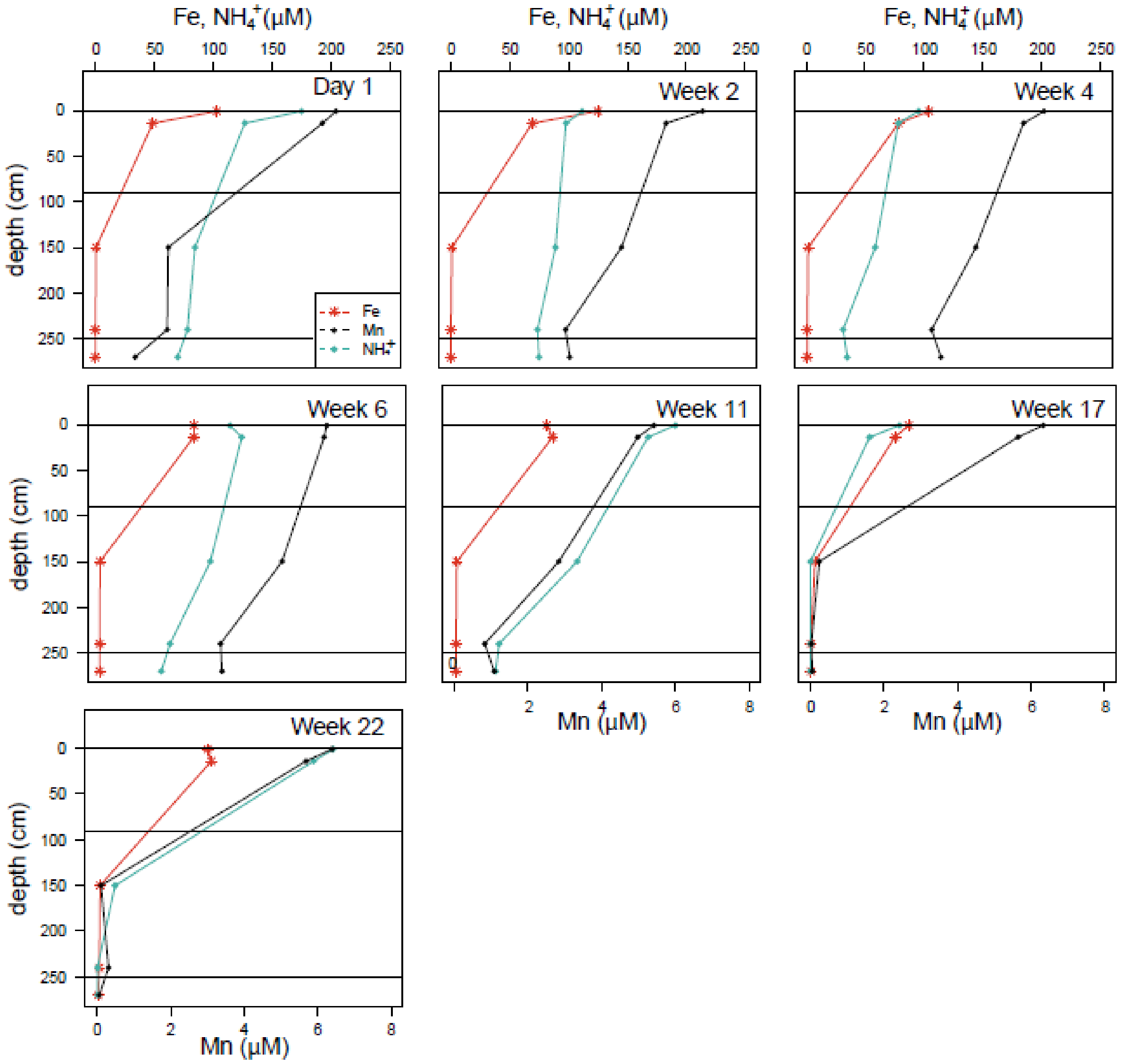

Figure S5. Temporal dynamics of dissolved iron (dFe, red stars), dissolved manganese (dMn, black diamonds), and ammonium (NH_4^+, light green diamonds) at 7 selected time points after inoculation of Filter 4. Further chemical data, including profiles of nitrite, nitrate, methane, oxygen, and pH are given in the supplementary data file.

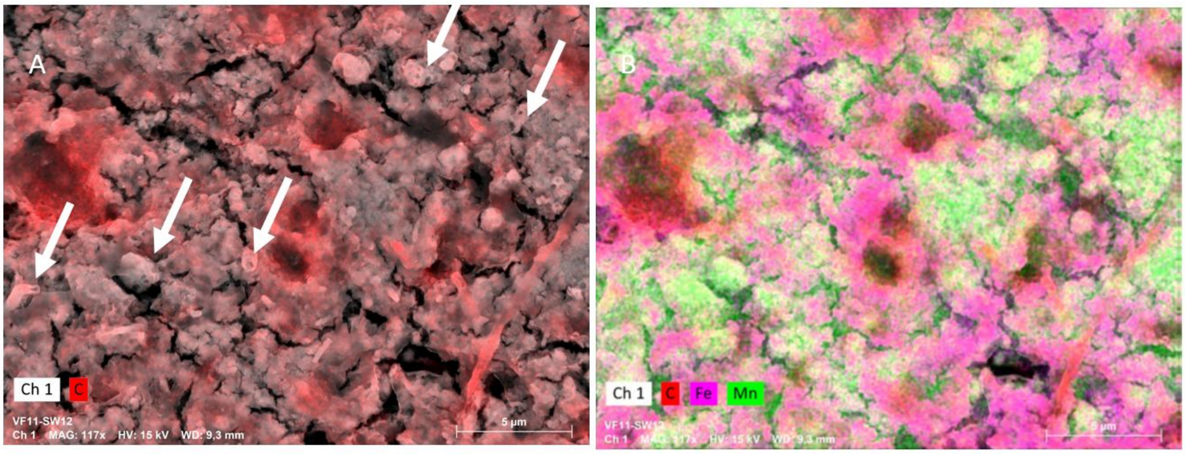

Figure S6. SEM-EDS images of filter material originating from the inoculum collected at 6 weeks at 125-150 cm depth from Filter 1 A) Arrows point to hollow tubes associated with biological iron oxidation. Carbon (C) in red; B) Elemental map showing the distribution of Fe and Mn oxides; carbon (C) is shown in red, iron (Fe) in pink, and manganese (Mn) in green.

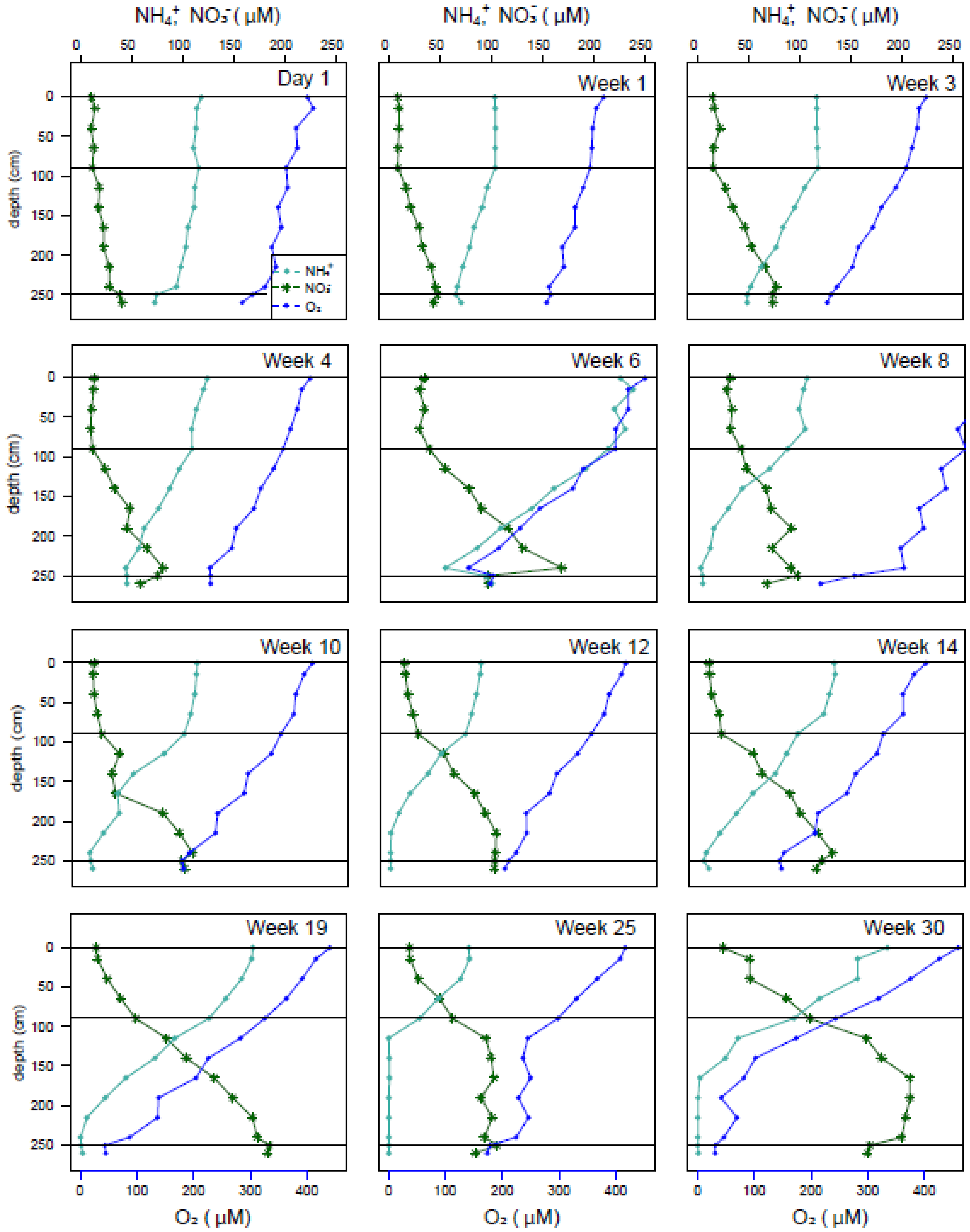

Figure S7. Temporal dynamics of ammonium (NH_4^+, light green diamonds), nitrate (NO_3^-, dark green stars), and oxygen (O_2, blue closed circles) at selected time points after inoculation of Filter 1.

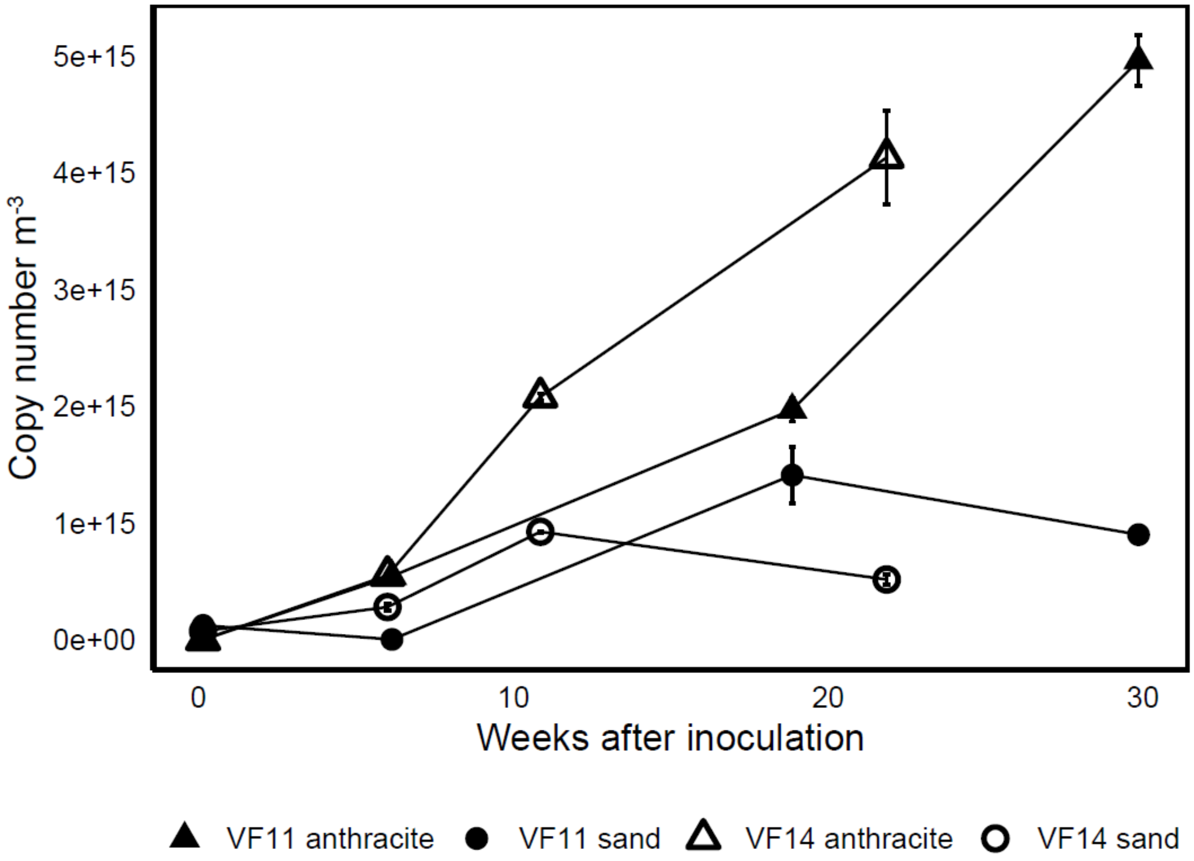

Figure S8. Absolute bacterial abundances. Copies of the 16S rRNA gene were quantified using qPCR for Filters 1 and 4 at different time points after inoculation. Error bars represent the standard deviation of technical triplicates.

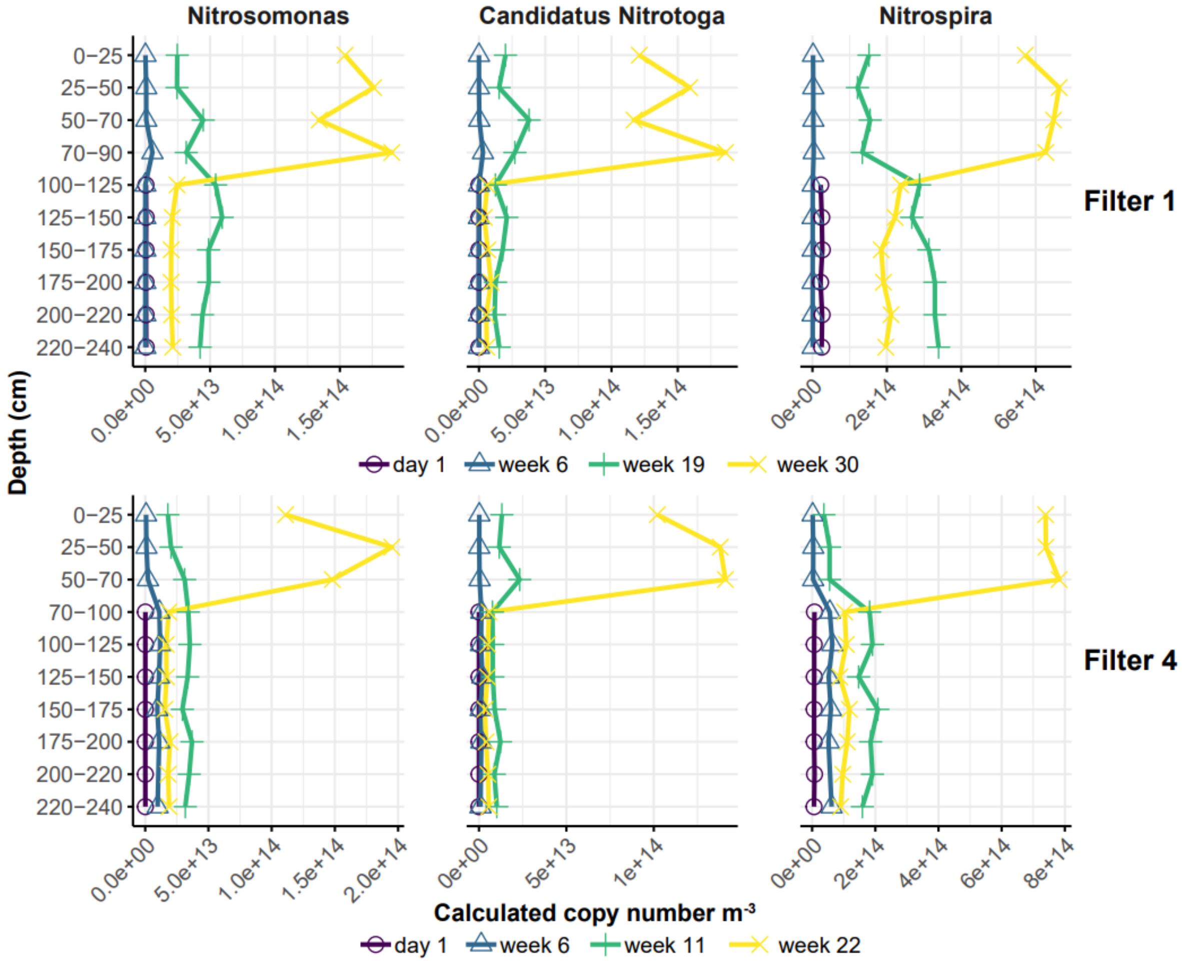

Figure S9. Calculated 16S rRNA gene copy numbers of the nitrifying community. Quantities of the nitrifying community of Filters 1 and 4 were calculated by combining the relative abundances based on 16S rRNA gene amplicon sequencing and qPCR.

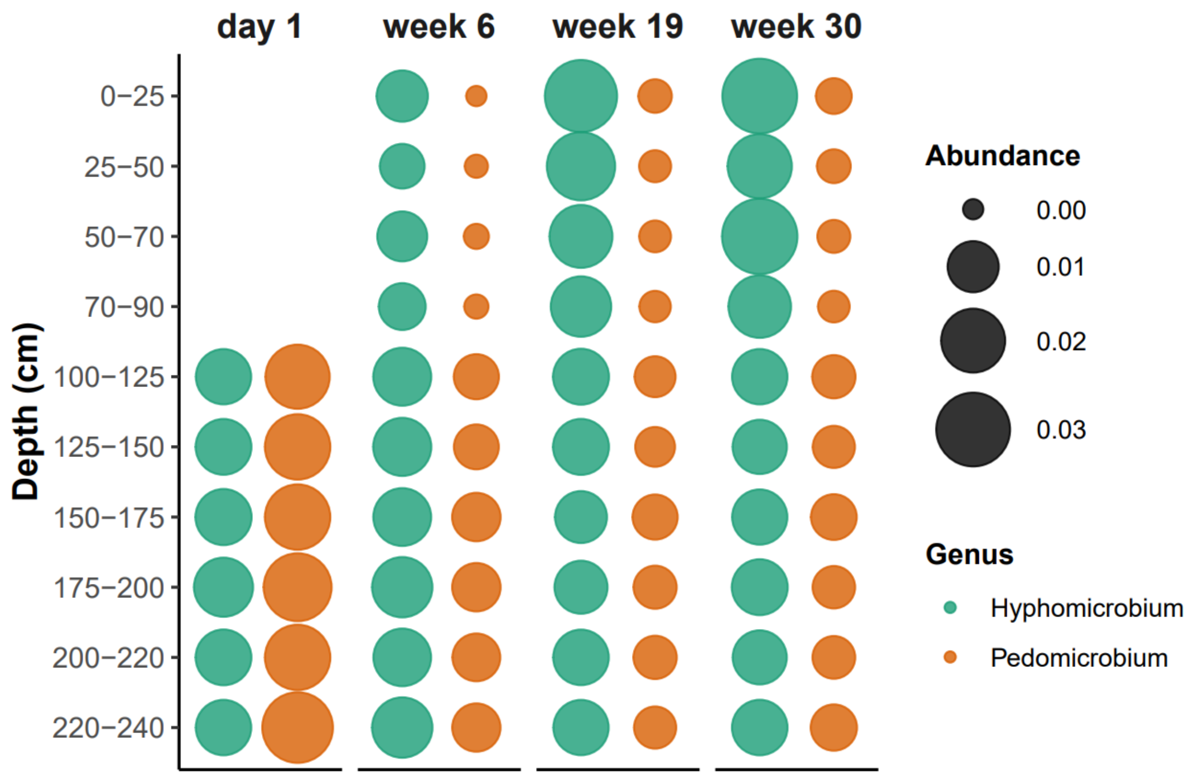

Figure S10. 16S rRNA gene amplicon sequencing-based relative abundances of *Hyphomicrobium* and *Pedomicrobium.*

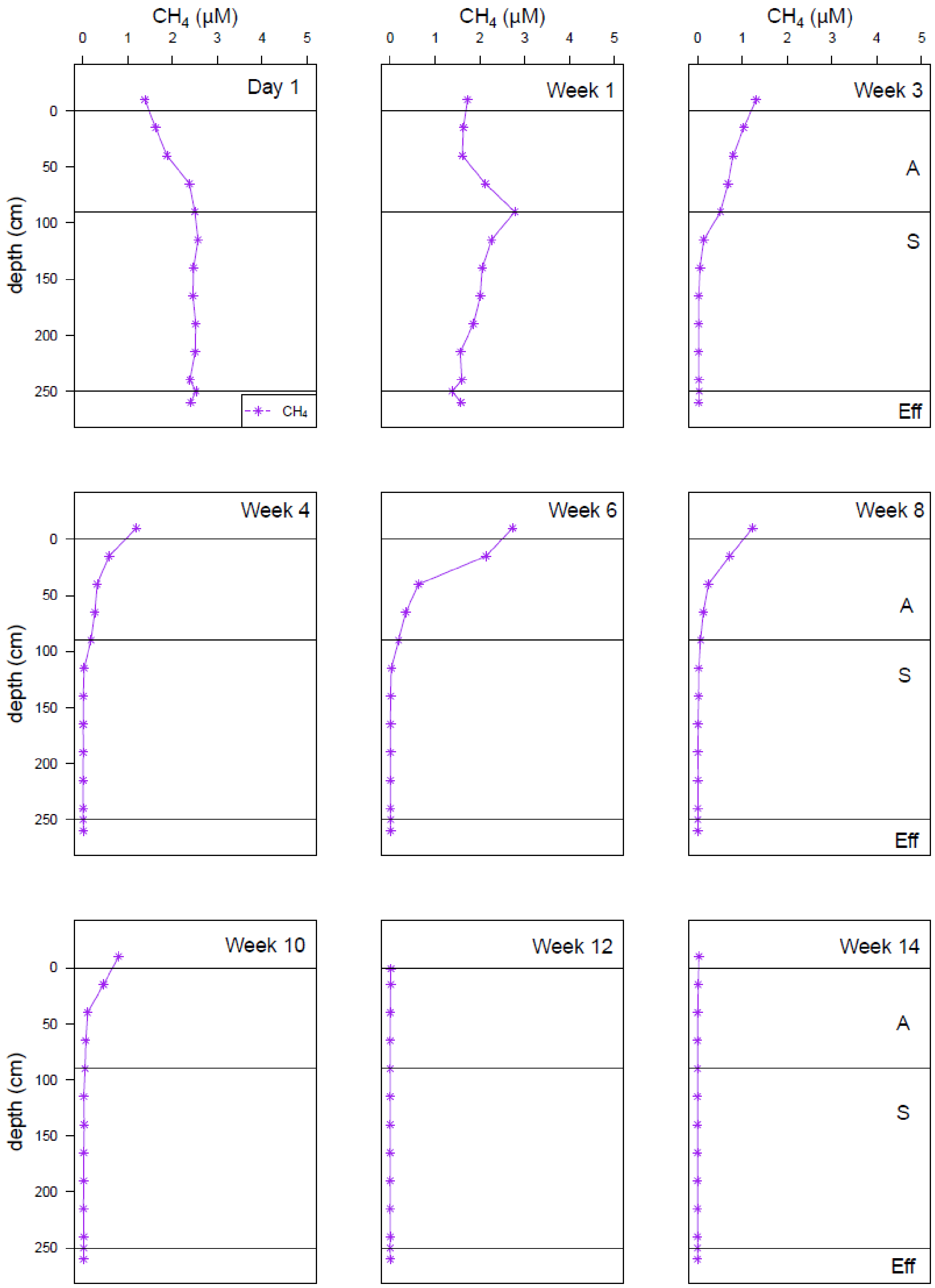

Figure S11. Temporal dynamics of methane (CH_4, purple stars) collected at 9 time points after inoculation of Filter 1.

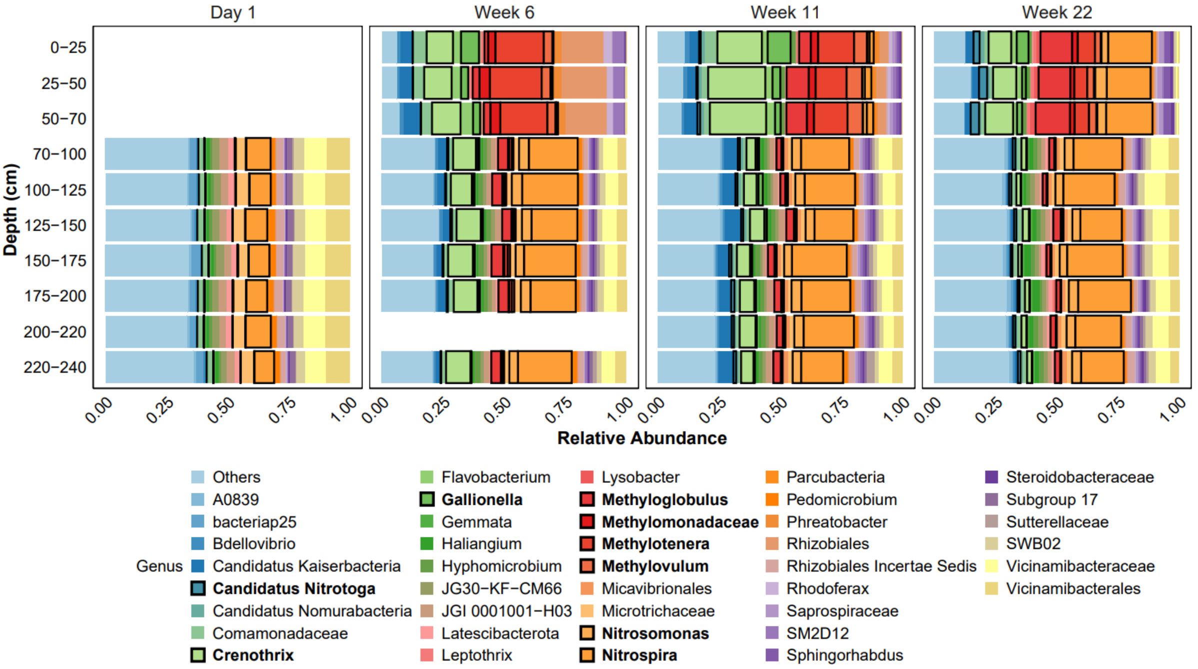

Figure S12. Microbial community composition of Filter 4 based on 16S rRNA gene amplicon sequencing data. Samples 1 day, 6, 11, and 22 weeks after filter replacement were analyzed. Highlighted are key players in ammonium, nitrite, methane, and iron removal: *Candidatus* Nitrotoga, *Crenothrix, Gallionella, Methyloglobulus, Methylomonadaceae, Methylotenera, Methylovulum, Nitrosomonas,* and *Nitrospira.*

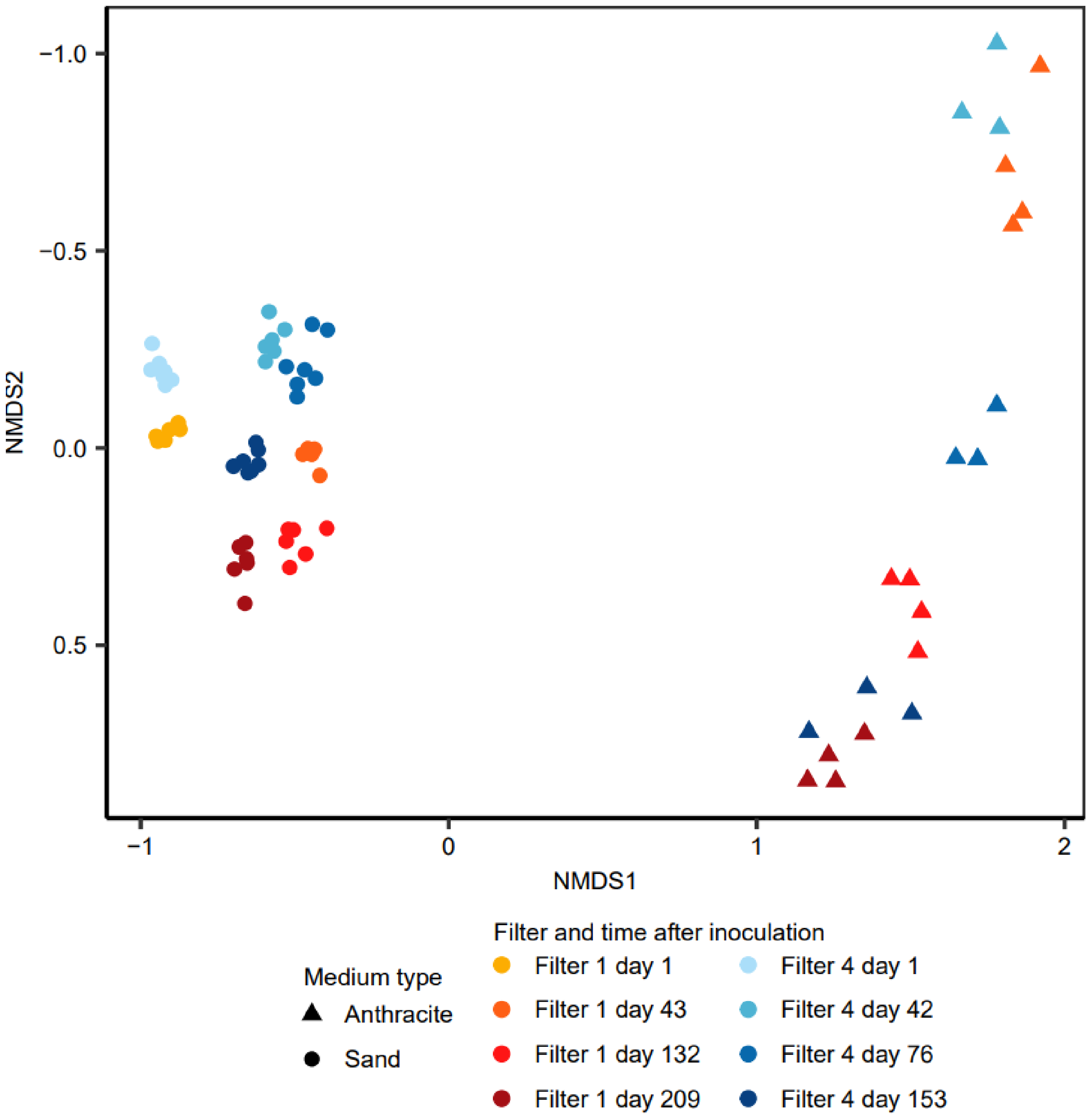

Figure S13. NMDS analysis of the beta diversity of the microbial communities. Beta diversity was calculated as Bray-Curtis dissimilarity for Filter 1 (red hues) and Filter 4 (blue hues), based on 16S rRNA gene amplicon sequencing data.

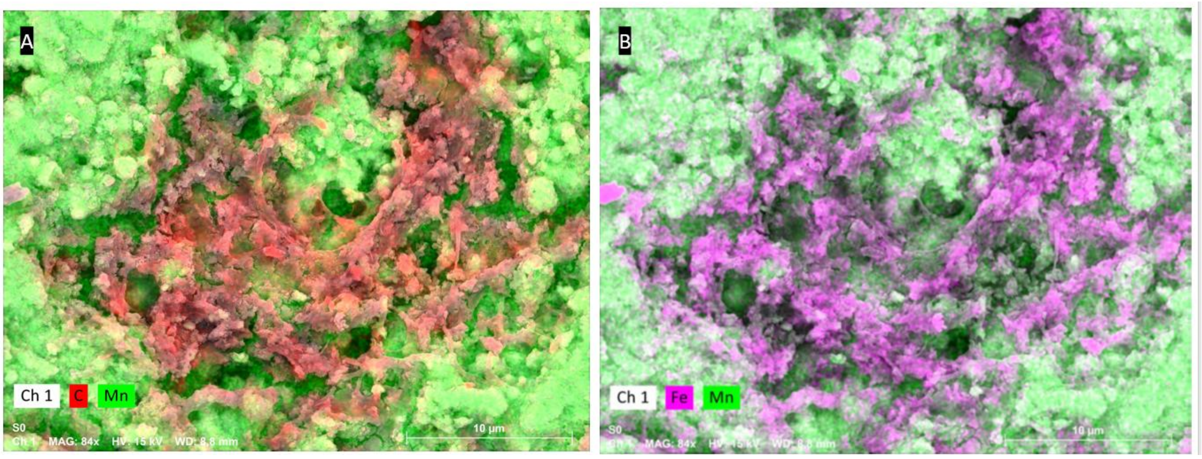

Figure S14. SEM-EDS images of filter inoculum (coated sand) collected at day 1 for Filter 1 A) Carbon (C) is shown in red, manganese oxides (Mn) in green. B) Manganese (Mn) is shown in green, iron (Fe) in purple. Note how iron and carbon are often associated together.

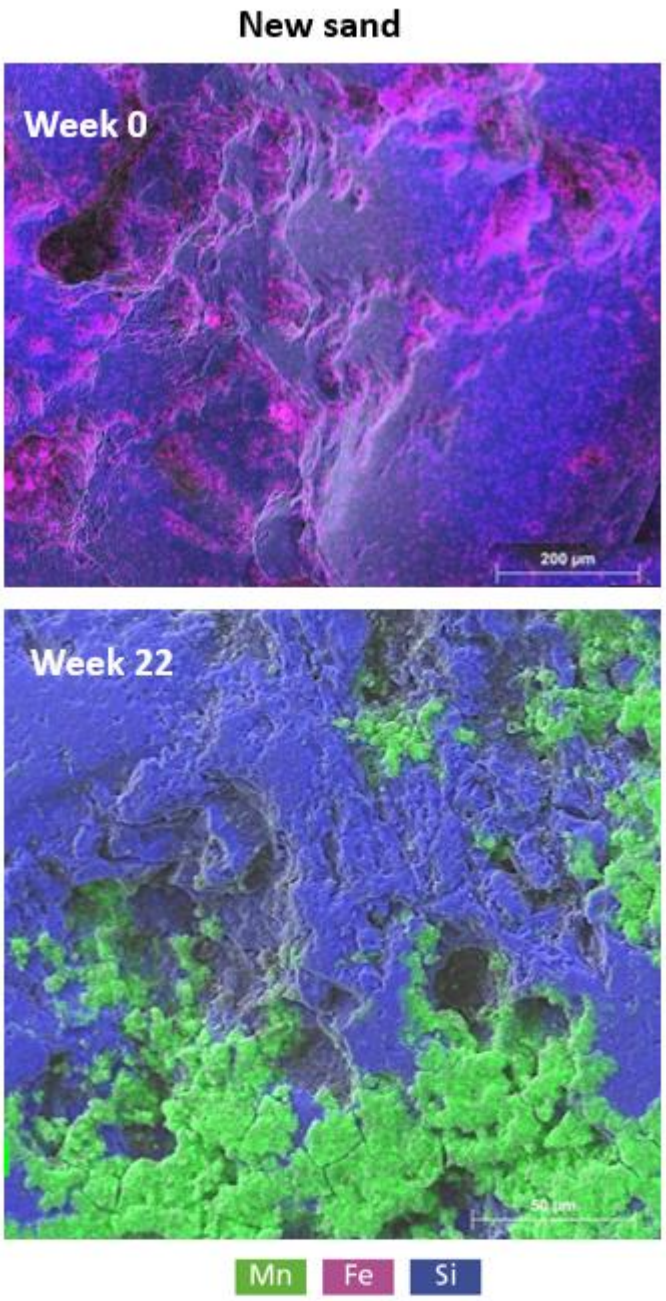

Figure S15. SEM-EDS images of filter medium coatings of new sand (125-150 cm depth) at day 1 (week 0) and week 22 from Filter 4. Iron (Fe) is shown in pink, manganese (Mn) in green, silica (Si) in blue.

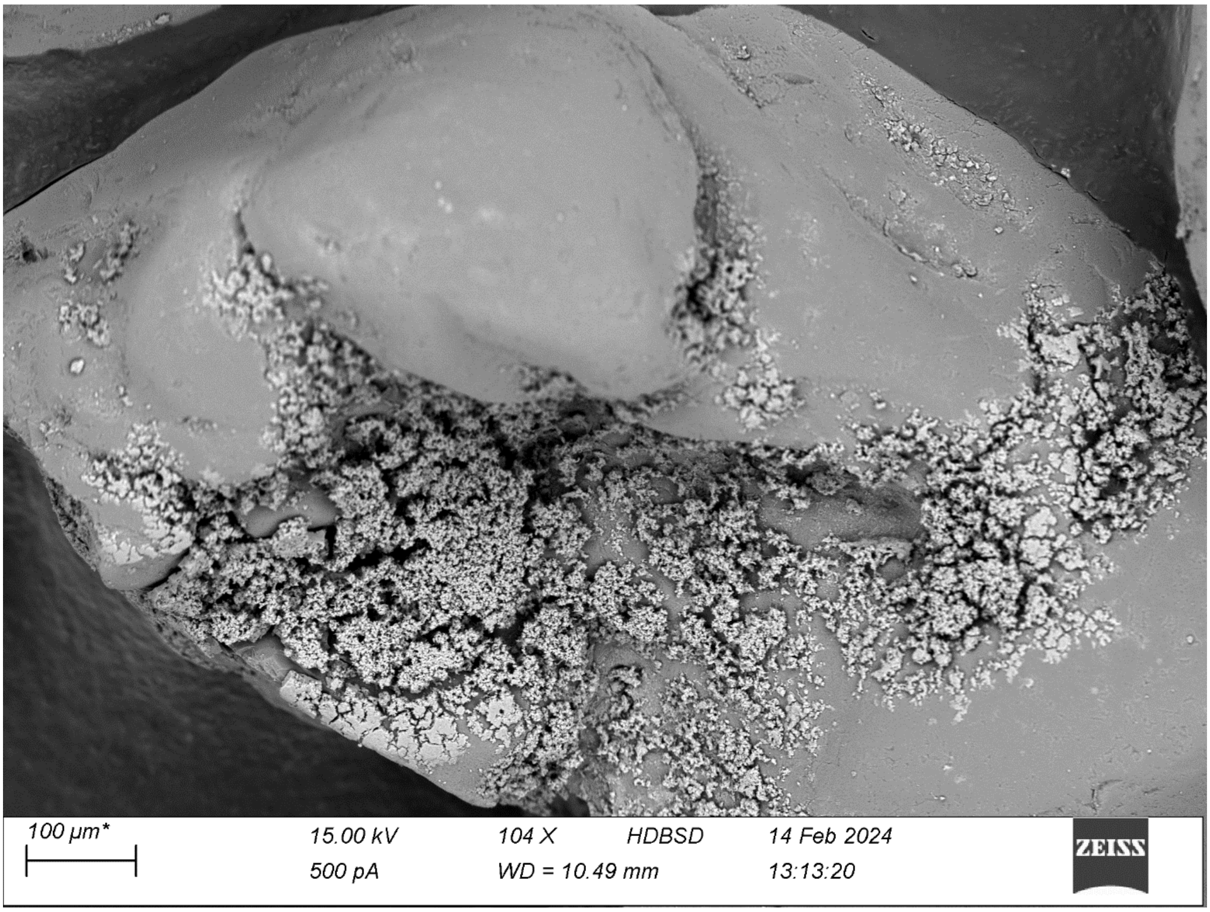

Figure S16. SEM image of fresh manganese oxides forming in the irregularities of the filter material collected in week 30 from Filter 1 at 125-150 cm depth.

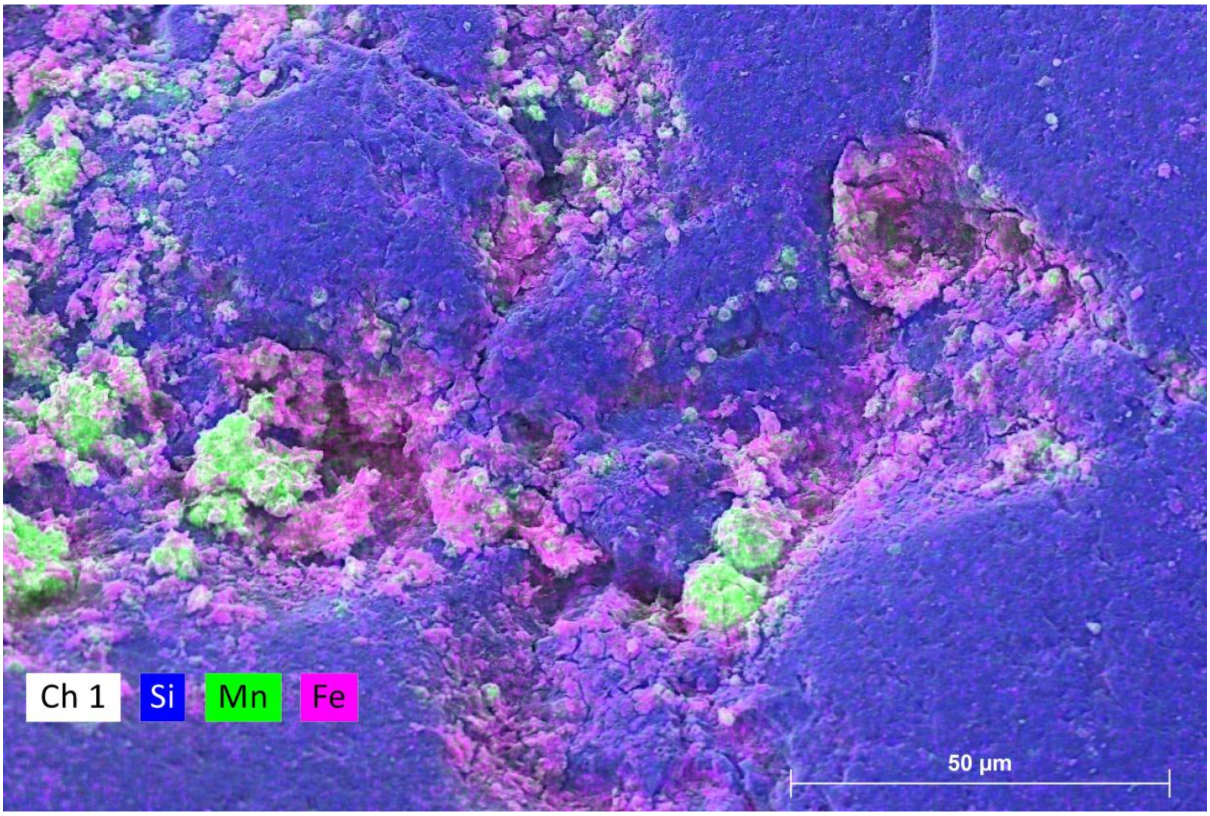

Figure S17. Fresh manganese oxides formed on iron oxide deposits in irregularities of the filter material collected in week 30 from Filter 1 at 125-150 cm depth. Iron (Fe) is shown in pink, manganese (Mn) in green, silica (Si) in blue.

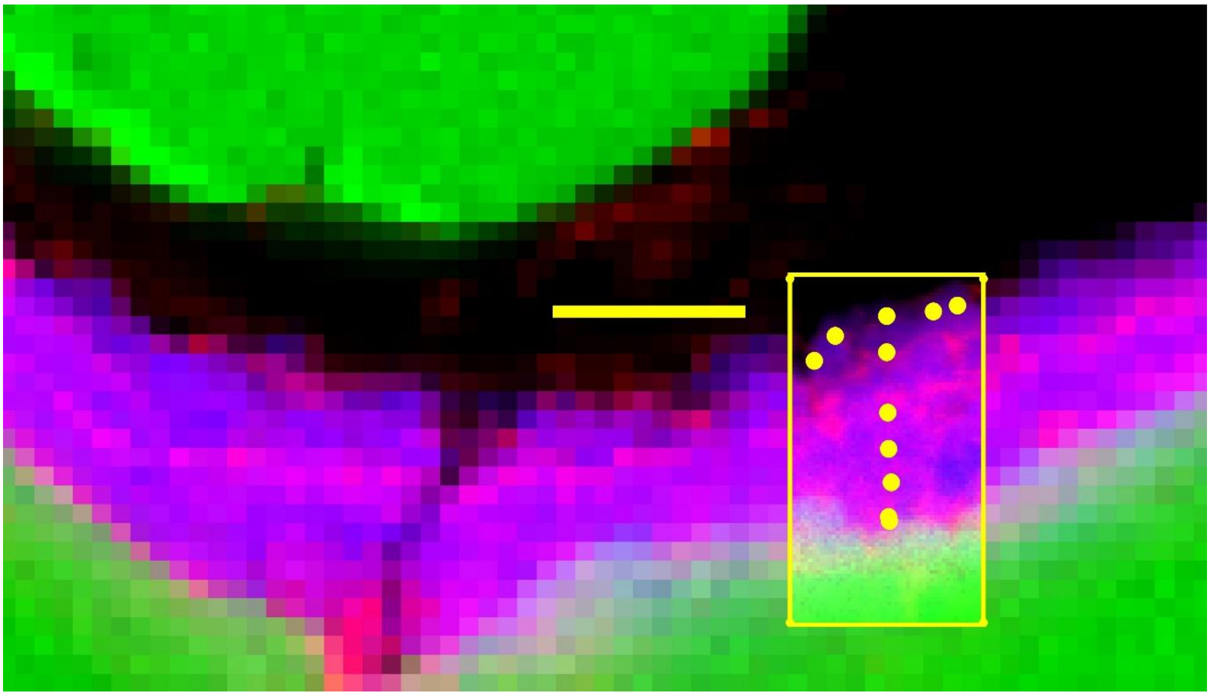

Figure S18. m-XRF map of a resin-embedded and polished section of an Mn-coated (bottom) and an uncoated (top) sand grain retrieved from Filter 1, collected after one day of operation, showing the elements Mn (blue), Fe (red), and Si (green). The scale bar represents a length of 50 mm. The large map was collected with a step size of 5 µm, while the map inside the yellow rectangle was mapped with a resolution of 0.5 µm. The yellow spots indicate positions at which XANES spectra were collected at the Mn K-edge.

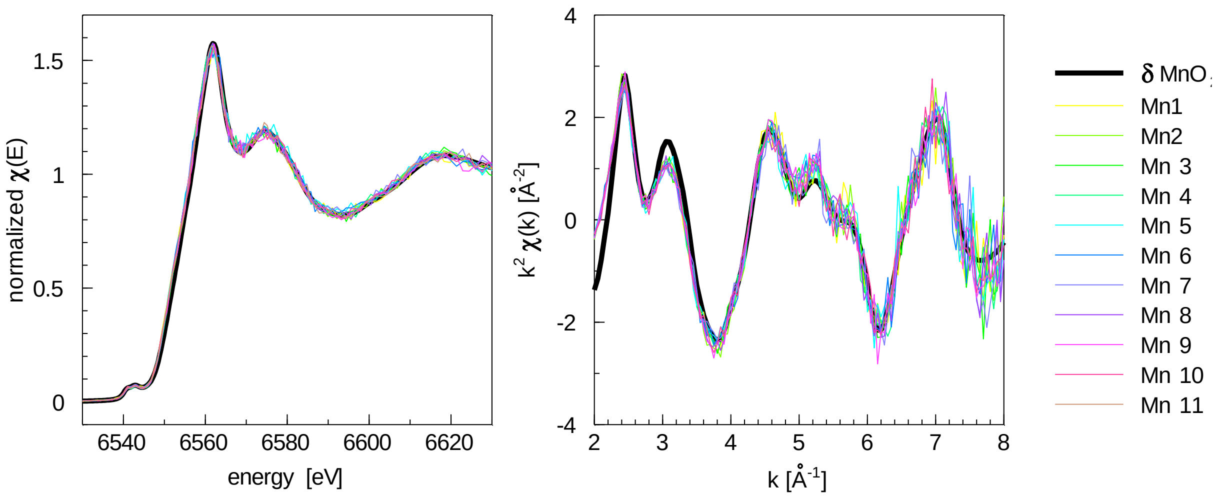

Figure S19. Self-absorption corrected Mn K-edge XANES (left) and k^2-weighted EXAFS (right) spectra across the coating of a sand grain retrieved from Filter 1, collected after one day in operation. The spectra are compared to the spectrum of d-MnO_2 reported by Webb et al., 2005 shifted by 0.4 eV. The corresponding positions of the spots are indicated in Figure 5.

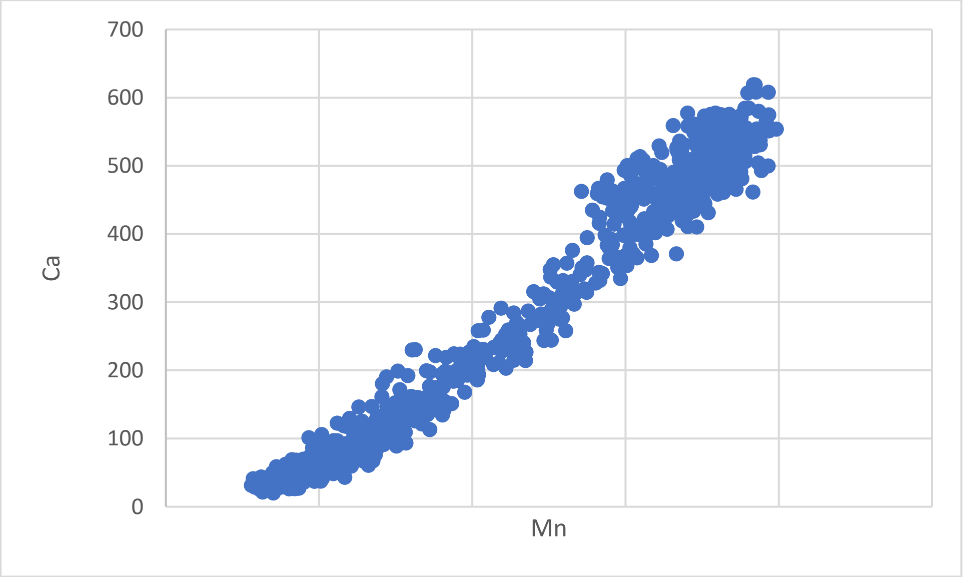

Figure S20. Correlation between Ca and Mn determined from spectra collected with µXRF from Filter 1.

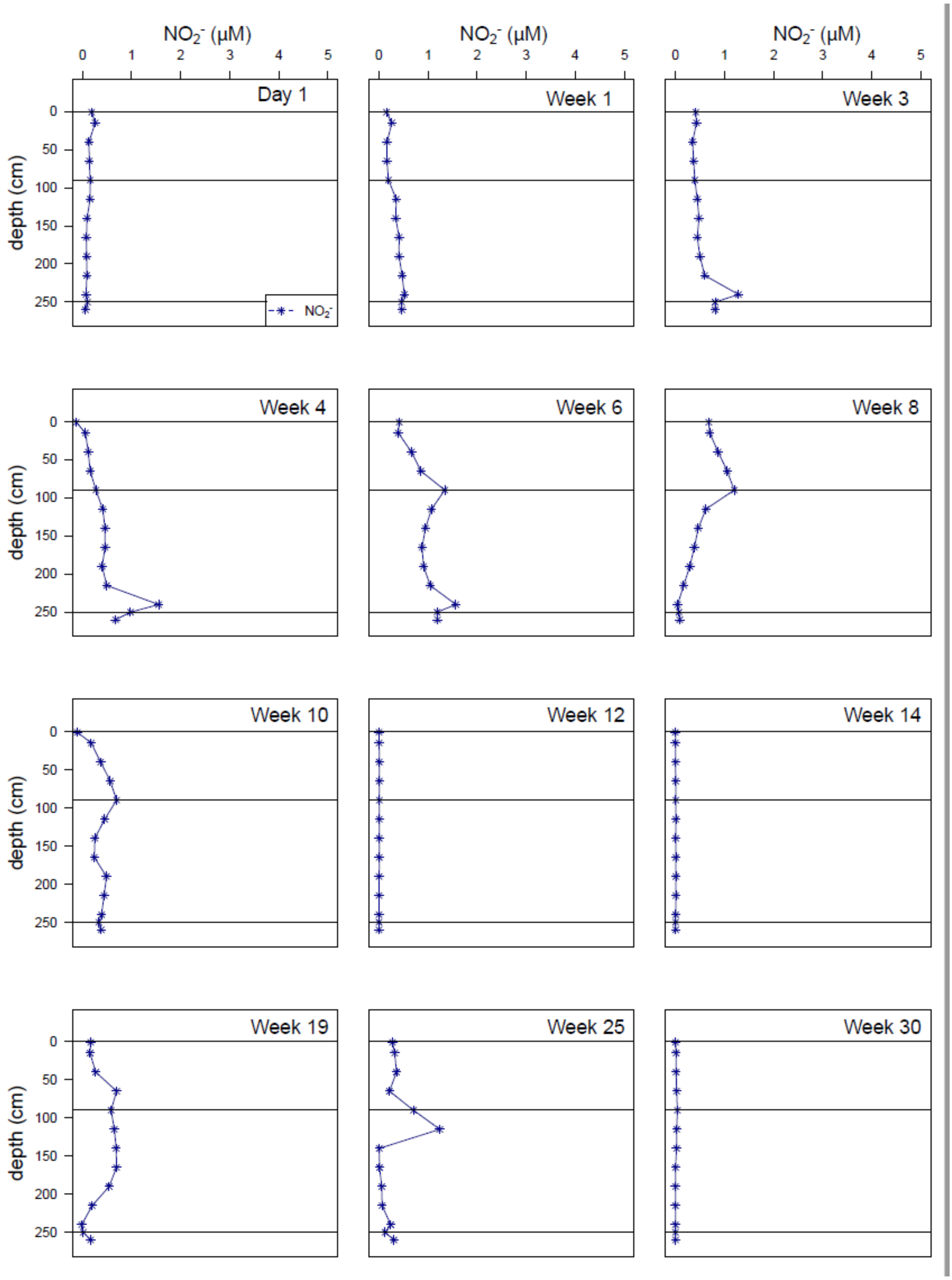

Figure S21. Temporal dynamics of nitrite (NO_2^-, blue stars) collected at 12 time points after inoculation of Filter 1.

Table S1. Calculated rates of ammonium removal per nitrifier cell in the filter at weeks 6, 19, and 30. A, anthracite; S, sand. #AOB, 16S rRNA copy numbers g^{-1} dw; NH_4^+ removal is given in mmol h^{-1} m^2 $filter^{-1}$; NH_4^+ removal per AOB cell is indicated in mmol h^{-1} 16S rRNA $copy^{-1}$.

				Relative to previous week	
	**Week 6**	**Week 19**	**Week 30**	**Week 19**	**Week 30**
#AOB (A)	3,54E+06	4,61E+07	2,40E+08	13	5,21
#AOB (S)	1,21E+06	3,31E+07	1,41E+07	27	0,43
Removal of NH_4^+ (A)	60	101	216	1,68	2,14
Removal of NH_4^+ (S)	271	298	225,6	1,10	0,76
total [mmol/h]	331	398	442	1,20	1,11
Removal per cell (A)	2,69E-11	3,47E-12	1,19E-12	0,13	0,34
Removal per cell (S)	9,26E-11	3,73E-12	6,64E-12	0,04	1,78

References:

Callahan, B. J., Mcmurdie, P. J., Rosen, M. J., Han, A. W., Johnson, A. J. A., & Holmes, S. P. (2016). *dada2: high-resolution sample inference from illumina amplicon data*. *13*(7). https://doi.org/10.1038/nMeth.3869

Caporaso, J. G., Lauber, C. L., Walters, W. A., Berg-Lyons, D., Huntley, J., Fierer, N., Owens, S. M., Betley, J., Fraser, L., Bauer, M., Gormley, N., Gilbert, J. A., Smith, G., & Knight, R. (2012). Ultra-high-throughput microbial community analysis on the Illumina HiSeq and MiSeq platforms. *The ISME Journal*, *6*, 1621–1624. https://doi.org/10.1038/ismej.2012.8

Herlemann, D. P., Labrenz, M., Jü Rgens, K., Bertilsson, S., Waniek, J. J., & Andersson, A. F. (2011). Transitions in bacterial communities along the 2000 km salinity gradient of the Baltic Sea. *The ISME Journal*, *5*, 1571–1579. https://doi.org/10.1038/ismej.2011.41

McMurdie, P. J., & Holmes, S. (2013). Phyloseq: An R Package for Reproducible Interactive Analysis and Graphics of Microbiome Census Data. *PLoS ONE*, *8*(4). https://doi.org/10.1371/journal.pone.0061217

Muyzer, G., De Waal,' And, E. C., & Uitierlinden2, A. G. (1993). Profiling of Complex Microbial Populations by Denaturing Gradient Gel Electrophoresis Analysis of Polymerase Chain Reaction-Amplified Genes Coding for 16S rRNA. *APPLIED AND ENVIRONMENTAL MICROBIOLOGY*, 695–700. https://journals.asm.org/journal/aem

Quast, C., Pruesse, E., Yilmaz, P., Gerken, J., Schweer, T., Yarza, P., Peplies, J., & Glöckner, F. O. (2013). The SILVA ribosomal RNA gene database project: Improved data processing and web-based tools. *Nucleic Acids Research*, *41*(D1), 590–596. https://doi.org/10.1093/nar/gks1219

Ravel, B., & Newville, M. (2005). ATHENA and ARTEMIS: Interactive graphical data analysis using IFEFFIT. *Physica Scripta T*, *T115*, 1007–1010. https://doi.org/10.1238/Physica.Topical.115a01007

Salomé, M., Cotte, M., Baker, R., Barrett, R., Benseny-Cases, N., Berruyer, G., Bugnazet, D., Castillo-Michel, H., Cornu, C., Fayard, B., Gagliardini, E., Hino, R., Morse, J., Papillon, E., Pouyet, E., Rivard, C., Solé, V. A., Susini, J., & Veronesi, G. (2013). The ID21 scanning X-ray microscope at ESRF. *Journal of Physics: Conference Series*, *425*(PART 18), 1–5. https://doi.org/10.1088/1742-6596/425/18/182004

Solé, V. A., Papillon, E., Cotte, M., Walter, P., & Susini, J. (2007). A multiplatform code for the analysis of energy-dispersive X-ray fluorescence spectra. *Spectrochimica Acta - Part B Atomic Spectroscopy*, *62*(1), 63–68. https://doi.org/10.1016/j.sab.2006.12.002

Terashima, S., Usui, A., & Imai, N. (1995). Two New Gsj Geochemical Reference Samples: Syenite Jsy-1 and Manganese Nodule Jmn-1. *Geostandards Newsletter*, *19*(2), 221–229. https://doi.org/10.1111/j.1751-908X.1995.tb00160.x

Webb, S. M., Tebo, B. M., & Bargar, J. R. (2005). Structural characterization of biogenic Mn oxides produced in seawater by the marine bacillus sp. strain SG-1. *American Mineralogist*, *90*(8–9), 1342–1357. https://doi.org/10.2138/am.2005.1669
